# Positive and negative regulation of transferred *nif* genes mediated by indigenous GlnR in Gram-positive *Paenibacillus polymyxa*

**DOI:** 10.1371/journal.pgen.1007629

**Published:** 2018-09-28

**Authors:** Tianshu Wang, Xiyun Zhao, Haowen Shi, Li Sun, Yongbin Li, Qin Li, Haowei Zhang, Sanfeng Chen, Jilun Li

**Affiliations:** State Key Laboratory for Agrobiotechnology, Key Laboratory of Soil Microbiology of Agriculture Ministry and College of Biological Sciences, China Agricultural University, Beijing, P. R. China; Universidad de Alicante, SPAIN

## Abstract

Ammonia is a major signal that regulates nitrogen fixation in most diazotrophs. Regulation of nitrogen fixation by ammonia in the Gram-negative diazotrophs is well-characterized. In these bacteria, this regulation occurs mainly at the level of *nif* (*ni*trogen *f*ixation) gene transcription, which requires a *nif*-specific activator, NifA. Although Gram-positive and diazotrophic *Paenibacilli* have been extensively used as a bacterial fertilizer in agriculture, how nitrogen fixation is regulated in response to nitrogen availability in these bacteria remains unclear. An indigenous GlnR and GlnR/TnrA-binding sites in the promoter region of the *nif* cluster are conserved in these strains, indicating the role of GlnR as a regulator of nitrogen fixation. In this study, we for the first time reveal that GlnR of *Paenibacillus polymyxa* WLY78 is essentially required for *nif* gene transcription under nitrogen limitation, whereas both GlnR and glutamine synthetase (GS) encoded by *glnA* within *glnRA* operon are required for repressing *nif* expression under excess nitrogen. Dimerization of GlnR is necessary for binding of GlnR to DNA. GlnR in *P*. *polymyxa* WLY78 exists in a mixture of dimers and monomers. The C-terminal region of GlnR monomer is an autoinhibitory domain that prevents GlnR from binding DNA. Two GlnR-biding sites flank the -35/-10 regions of the *nif* promoter of the *nif* operon (*nifBHDKENXhesAnifV*). The GlnR-binding site Ⅰ (located upstream of -35/-10 regions of the *nif* promoter) is specially required for activating *nif* transcription, while GlnR-binding siteⅡ (located downstream of -35/-10 regions of the *nif* promoter) is for repressing *nif* expression. Under nitrogen limitation, GlnR dimer binds to GlnR-binding siteⅠ in a weak and transient association way and then activates *nif* transcription. During excess nitrogen, glutamine binds to and feedback inhibits GS by forming the complex FBI-GS. The FBI-GS interacts with the C-terminal domain of GlnR and stabilizes the binding affinity of GlnR to GlnR-binding site Ⅱ and thus represses *nif* transcription.

## Author summary

GlnR is a global transcription regulator of nitrogen metabolism in *Bacillus* and other Gram-positive bacteria. GlnR generally functions as repressor and inhibits gene transcription under excess nitrogen. Our study for the first time reveals that GlnR simultaneously acted as an activator and a repressor for nitrogen fixation of *Paenibacillus* by binding to different loci of the single *nif* promoter region according to nitrogen availability. In excess glutamine, the feedback inhibited form of glutamine synthetase (GS) encoded by *glnA* within *glnRA* operon directly interacts with the C-terminal domain of GlnR and then controls the GlnR activity. Also, overexpression of *glnR* or deletion of *glnA* or mutagenesis of GlnR-binding site Ⅱ led to constitutive *nif* expression in the absence or presence of high (100 mM) concentration of ammonia. This work represents the first instance of a dual positive and negative regulatory mechanism of nitrogen fixation.

## Introduction

Biological nitrogen fixation, the conversion of atmospheric N_2_ to ammonia (NH_3_), is carried out by a specialized group of prokaryotes and plays an important role in world agriculture [1]. Yet the great demands for nitrogen in modern agriculture far outstrip this source of fixed nitrogen, and chemical nitrogen (N) fertilizer is used extensively in agriculture. Overuses of N fertilizer in many parts of the world have led to soil, water, and air pollution [2].

Ammonia is a major signal that regulates nitrogen fixation in most diazotrophs [3, 4]. Regulation of nitrogen fixation in the Gram-negative diazotrophs is well-characterized. In these bacteria, this regulation occurs mainly at the level of *nif* gene transcription, which requires a *nif*-specific activator, NifA [5]. NifA acts as an enhancer binding protein (EBP) that recognizes sequences (TGT-N10-ACA), located upstream of the -24/-12 region of the promoters controlled by RNA polymerase containing the alternative σ^54^ factor [3,6–8].

*Paenibacillus* is a large genus of Gram-positive, facultative anaerobic, endospore-forming bacteria. The genus *Paenibacillus* currently comprises more than 150 named species, approximately 20 of which have nitrogen fixation ability, including eight novel species described by our laboratory [9]. Diazotrophic Paenibacilli has been extensively used as a bacterial fertilizer in agriculture [10]. However, the regulation mechanism of nitrogen fixation in response to nitrogen availability in Paenibacilli is not clarified, partially due to hardness in genetic transformation of these bacteria. Our recent studies by comparative genomic sequence analysis have revealed that a minimal and compact *nif* cluster comprising nine genes (*nifB nifH nifD nifK nifE nifN nifX hesA nifV*) encoding Mo-nitrogenase is conserved in 15 N_2_-fixing *Paenibacillus* strains [11]. Phylogeny analysis suggests that the ancestral *Paenibacillus* did not fix nitrogen. The N_2_-fixing *Paenibacillus* strains were generated by acquiring the *nif* cluster via horizontal gene transfer (HGT) from a source related to Frankia [11]. The 9 genes (*nifBHDKENXhesAnifV*) within the *nif* cluster are organized as an operon under control of a σ^A^ (σ^70^)-dependent promoter located in front of *nifB* gene [12]. A global transcriptional profiling analysis revealed that *nif* gene transcription in *P*. *polymyxa* WLY78 was strongly regulated by ammonium and oxygen [13]. However, unlike Gram-negative diazotrophs, diazotrophic Paenibacilli have no *nifA* gene encoding transcriptional activator NifA and no NifA-binding site in the *nif* promoter region. But a *glnR* gene and GlnR/TnrA-binding sites in the promoter region of the *nif* operon are conserved in the 15 diazotrophic *Paenibacillus* strains by comparative genomics analyses [11], indicating the role of GlnR as a regulator of nitrogen fixation. GlnR is a central regulator of nitrogen metabolism in the class Bacilli, and the *glnR* gene in the diazotrophic Paenibacilli is not associated with the transferred *nif* gene cluster, indicating that *Paenibacillus* GlnR is indigenous. The recent studies with Surface Plasmon Resonance (SPR) experiments have demonstrated that GS stabilizes the binding of GlnR to nitrogen fixation gene operators in *Paenibacillus riograndensis* SBR5 [14]. However, these studies did not fully investigate the regulatory mechanism of GlnR in nitrogen fixation.

GlnR and TnrA are the two transcriptional regulators for the regulation of nitrogen metabolism in the Gram-positive model organism *Bacillus subtilis* [15,16]. They were previously recognized as the members of the MerR family regulators according to their common winged-HTH (helix-turn-helix) domains [17]. However, the recent studies have revealed that TnrA and GlnR are a new family of dimeric DNA-binding proteins with C-terminal, flexible, effector-binding sensors that modulate their dimerization that represents a separate branch of the MerR family proteins [18]. TnrA/GlnR form weak dimers by hydrophobic residues located on its winged-HTH and residues in its N-terminal helix [18], whereas MerR proteins form tight dimers via their extended C-terminal coiled coils [19]. Both of GlnR and TnrA proteins of *B*. *subtilis* have a high sequence similarity at their N terminal domains and bind a common consensus sequence (5’-TGTNAN7TNACA-3’), but the C terminal domains of these proteins differ completely [20–24]. GlnR of *B*. *subtilis* generally acts as a repressor repressing gene or operons required for ammonium assimilation like the *glnRA* operon, *tnrA* and *ureABC* (the urease gene cluster) under nitrogen-excess condition [15,16,25]. In contrast, TnrA serves in most cases as an activator, for instance activating ammonia transport (*nrgAB* = *amtBglnK*), *ureABC*, nitrate and nitrite reduction (*nasABCDEF*) and its own gene (*tnrA*) [23], whereas in a few cases, it acts like GlnR as a repressor repressing *alsT* (encoding an H^+^/Na^+^ amino acid symporter)[26], *gltAB* (encoding glutamate synthase)[27,28] and *ilvBHC-leuABCD* (encoding branched-chain amino acid biosynthesis proteins)[29]. During excess nitrogen, glutamine (Gln) binds to and feedback inhibits glutamine synthetase (GS, the product of *glnA*)) by forming the complex FBI-GS. Formation of the feedback-inhibited GS (FBI-GS) signals the presence of excess nitrogen and transmits that signal by interacting with and affecting the DNA-binding and transcription programs of both GlnR and TnrA. Under nitrogen limitation, the C-terminal region of GlnR folds back and forms an autoinhibitory helix that prevents dimer formation and thus inhibits DNA binding [18,20–22]. Under excess nitrogen, FBI-GS functions as a chaperone by a transient interaction with the GlnR autoinhibitory domain and relieves autoinhibition, shifting the equilibrium from the inhibited form to the DNA-binding active form and thus turning on GlnR repression [18,20–22]. In contrast, FBI-GS forms a stable complex with TnrA, inhibiting its DNA-binding function under excess nitrogen, whereas TnrA is released from FBI-GS, allowing TnrA dimerization and activation of its transcription program under nitrogen limitatiom. GlnK appears to play an ancillary role in TnrA dimerization by acting as a templating agent for TnrA [25–30].

In this study, we fully investigate the regulation mechanisms of nitrogen fixation in *P*. *polymyxa* WLY78 by using comprehensive molecular methods. We reveal that during nitrogen limitation, GlnR binds to GlnR-binding site Ⅰ located upstream of -35/-10 regions of *nif* promoter of *nif* operon (*nifBHDKENXhesAnifV*) in a weak and transient association way and then activates *nif* transcription. During excess nitrogen, glutamine (Gln) binds to and feedback inhibits glutamine synthetase (GS) by forming the complex FBI-GS. FBI-GS interacts with C-terminal domain of GlnR and stabilizes the binding of GlnR to site Ⅱ located downstream of *nifB* transcription start codon and thus represses *nif* transcription. GS encoded by *glnA* within *glnRA* operon is involved in regulation of *nif* transcription. Also, overexpression of *glnR* and mutagenesis of *glnA* or GlnR-binding site Ⅱ led to constitutive nitrogen fixation in the absence or presence of ammonia. Our study not only reveals the novel regulation mechanisms of *nif* gene expression in Paenibacilli, but also provides insight into dual active and repressive functions of GlnR.

## Results

### GlnR is essentially required for nitrogen fixation under nitrogen limitation

The genome of *P*. *polymyxa* WLY78 contains a *glnR* gene and two paralogs of *glnA*, but it lacks a *tnrA* gene [11]. We found that of the two *glnA* genes, one was linked to *glnR* as a dicistronic *glnRA* operon and the other (here designated as *glnA1*) was elsewhere in the genome. The current analysis by using BLAST alignment showed that GS and GS1 proteins encoded by the *glnA* and *glnA1* genes had 39% identity.

**Fig 1:**
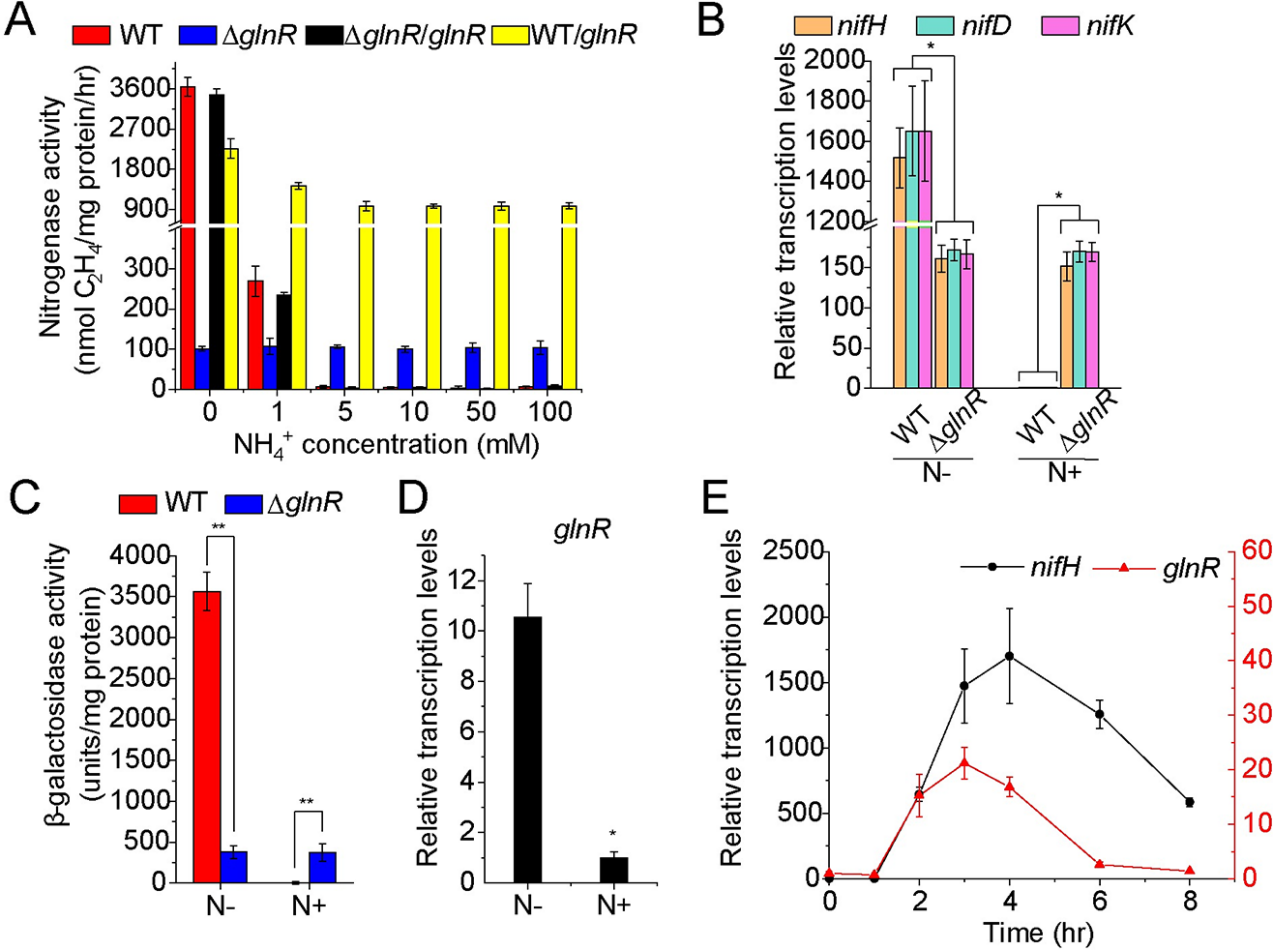
GlnR controls the nitrogenase activity and *nif* transcription in *P*. *polymyxa* WLY78. **A.** Nitrogenase activities of WT (the wild-type), Δ*glnR* (deletion mutant), Δ*glnR/glnR* (complementation strain) and WT/*glnR* (overexpression strain). These strains were grown anaerobically in nitrogen-deficient medium containing 2 mM glutamate supplemented with different concentration of NH_4_Cl at 0, 1, 5, 10, 50 and 100 mM. The nitrogenase activities of these strains were assayed by C_2_H_4_ reduction method and expressed at nmol C_2_H_4_/mg protein/hr. **B.** qRT-PCR analysis of the relative mRNA levels of the *nifHDK* genes in the WT and Δ*glnR* strains grown in nitrogen-limited and -excess media. N-: nitrogen-limited condition (2 mM glutamate as the only nitrogen source). N+: nitrogen-excess condition (2 mM glutamate + 100 mM NH_4_^+^). The relative expression level was calculated using ΔΔCt method.The transcription levels of genes in the WT strain under nitrogen-excess condition were arbitrarily set to 1.0. **C.** β-galactosidase activity of a P*nif-lacZ* fusion in the WT and Δ*glnR* strains grown in nitrogen-limited (N-) and -excess (N+) conditions. **D.** qRT-PCR analysis of the relative transcription levels of *glnR* gene under nitrogen-limited (N-) and -excess (N+) conditions. The transcription levels of *glnR* gene under nitrogen-excess condition were arbitrarily set to 1.0. **E.** qRT-PCR analysis of the transcription profiles of *glnR* and *nifH* under nitrogen limitation. The transcription levels of genes at time 0 hr were arbitrarily set to 1.0. Results are representative of at least three independent experiments. Error bars indicate SD. **P < 0.01; *P < 0.05.

To elucidate the function of GlnR in nitrogen fixation of *Paenibacillus*, we constructed an in-frame deletion mutant Δ*glnR*, a complemention strain (Δ*glnR/glnR*) for the mutated *glnR* and an overexpression strain (WT/*glnR*), as described in S1 Fig. In comparison with wild-type *P*. *polymyxa* WLY78 which exhibited the highest nitrogenase activity in the absence of NH_4_^+^ and no activity in the presence of more than 5 mM NH_4_^+^, activity in Δ*glnR* mutant was at basal constitutive levels under all conditions (Fig 1A). Deletion of *glnR* resulted to nearly loss of activity, indicating that GlnR is essentially required for nitrogen fixation under nitrogen limitation. Somewhat higher activity was observed in the Δ*glnR* mutant at high ammonia than in the ammonia-repressed wild-type strain. Complementation of Δ*glnR* with a single copy of *glnR* integrated on the *amyE* site of its genome restored nitrogenase activity to the wild-type level in complemented strain (Δ*glnR/glnR*), suggesting that change of nitrogenase activity was due solely to deletion of *glnR*. Overexpression of *glnR* by introduction of *glnR* carried on multicopy vector pHY300PLK into wild-type strain led to enhancement of activity in the presence of NH_4_^+^. The Δ*glnR* and the wild-type strains exhibited similar growth phenotypes on minimal media with glutamine, glutamate and ammonium as sole nitrogen sources (S2 Fig). Taken together, these results indicate for the first time that GlnR positively regulates nitrogen fixation under nitrogen-limited condition.

To examine the effect of *glnR* mutation on the transcription of *nif* genes in *P*. *polymyxa* WLY78, the transcription levels of *nifH*, *nifD* and *nifK* were determined by qRT-PCR. As shown in Fig 1B, the transcription levels of the *nifHDK* in wild-type strain exhibited more than 1000-fold of increase under nitrogen-limited condition (2 mM glutamate as sole nitrogen) compared to nitrogen-excess condition (2 mM glutamate + 100 mM NH_4_^+^). However, the *nifHDK* genes in Δ*glnR* mutant were expressed constitutively under both conditions at very low level which was approximately 2.7% of that observed in wild-type strain under nitrogen-limited condition (Fig 1B). These results are consistent with nitrogenase activity in this Δ*glnR* mutant, indicating that GlnR activates *nif* transcription under nitrogen-limited condition.

To further examine the effect of GlnR on regulation of *nif* expression, a transcriptional *lacZ* fusion to *nif* promoter region was constructed and then this P*nif-lacZ* fusion was introduced into wild-type and Δ*glnR* mutant, respectively. As shown in Fig 1C, the β-galactosidase levels produced by P*nif-lacZ* fusion in wild-type strain were 5000-fold higher in nitrogen-limited condition than in nitrogen-excess condition. However, the β-galactosidase levels produced by P*nif-lacZ* fusion in Δ*glnR* mutant were similar in both conditions. The data are consistent with the above described qRT-PCR results and nitrogenase activities.

Furthermore, qRT-PCR analysis demonstrated that *glnR* transcription was highly induced under nitrogen-limited condition compared to under nitrogen-excess condition (Fig 1D), suggesting that *glnR* expression itselfis nitrogen-dependent. Also, the transcription profiles of *glnR* and *nifH* were similar under nitrogen limitation (Fig 1E). The current results are consistent with our previous global transcriptional profiling analysis that the expressions of *glnR* and *nif* genes were significantly up-regulated when *P*. *polymyxa* WLY78 was grown in N_2_-fixing condition (without O_2_ and NH_4_^+^) compared to non-N_2_-fixing condition (air and 100 mM NH_4_^+^) [13]. These results indicate that the expressions of *glnR* and *nif* genes are highly coordinated.

### Both GS and GlnR are required for negative regulation of nitrogen fixation

**Fig 2:**
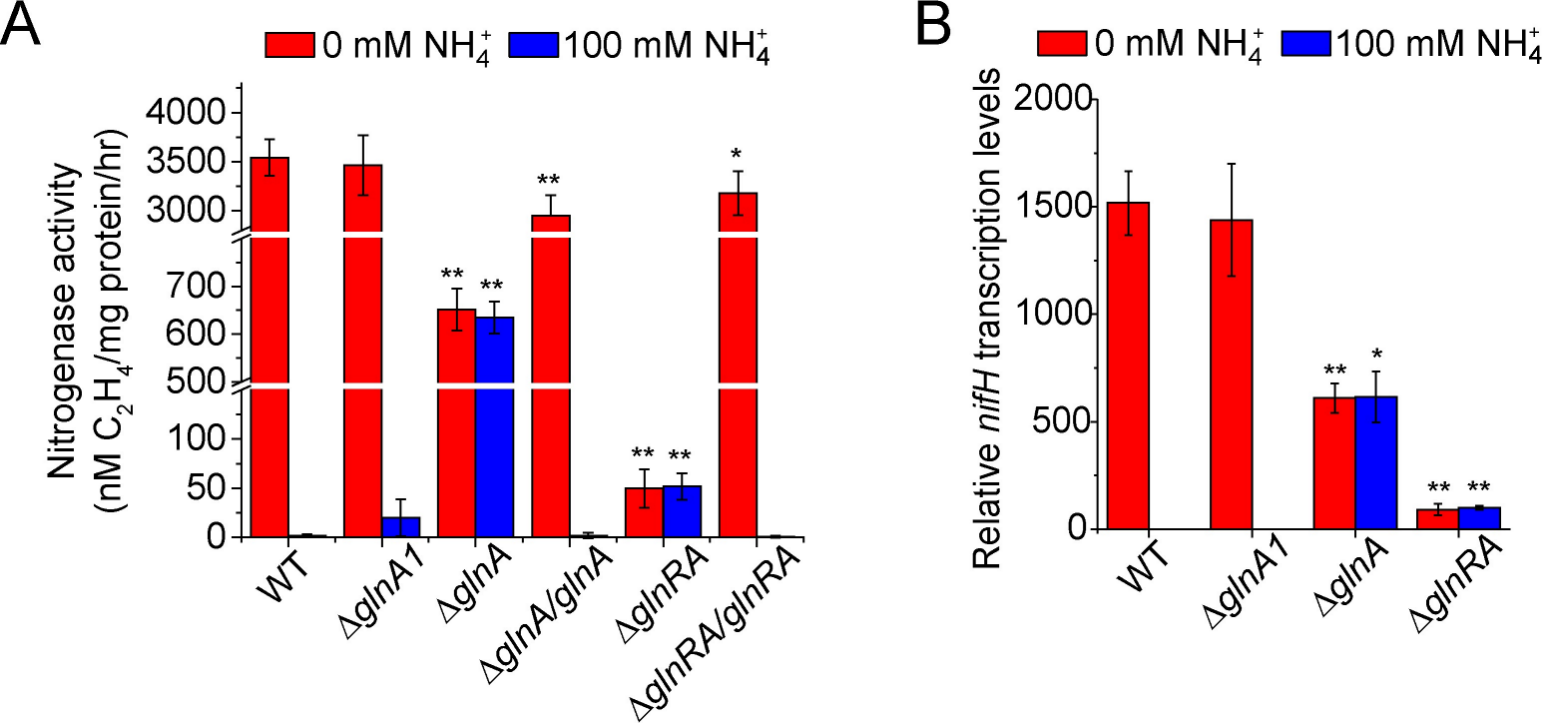
The functions of GlnR and GS proteins in repressing nitrogenase activity and *nif* expression. **A.** Nitrogenase activities of WT, deletion mutants Δ*glnA1*, Δ*glnA* and Δ*glnRA* and complementary strains Δ*glnA/glnA* and Δ*glnRA/ glnRA* under nitrogen-limited and -excess conditions. **B.** qRT-PCR analysis of the relative transcription levels of the *nifH* gene in different strains under both nitrogen-limited and–excess conditions. The transcription levels of *nifH* in the WT strain under nitrogen-excess condition were arbitrarily set to 1.0. Results are representative of at least three independent experiments. Error bars indicate SD. **P < 0.01; *P < 0.05.

To examine the role of GS proteins encoded by *glnA* and *glnA1* in regulation of nitrogen fixation, a series of in-frame-deletion mutants, including Δ*glnA1*, Δ*glnA* and Δ*glnRA* mutants, and their complementary strains Δ*glnA/glnA* and Δ*glnRA/glnRA* were constructed as described in S1 Fig. We found that nitrogenase activities were similar in Δ*glnA1* mutant and wild-type strain under both nitrogen-limited and -excess conditions, suggesting that *glnA1* is not involved in regulation of nitrogen fixation (Fig 2A). However, nitrogenase activity in Δ*glnA* mutant was produced constitutively at modest level under both nitrogen-limited and -excess conditions. Complementation of Δ*glnA* with *glnA* gene (complementary strain Δ*glnA/glnA*) restored the nitrogenase activity to the wild-type level (basal nitrogenase activity) under nitrogen-excess condition and to 80% of wild-type level (high nitrogenase activity) under nitrogen-limited condition (Fig 2A). Nitrogenase activity in Δ*glnRA* double mutant was almost abolished just as observed in Δ*glnR* single mutant. Complementation study showed that *glnRA* could partially restored the activity of Δ*glnRA* double mutant (Fig 2A), suggesting that the role of GS is dependent on GlnR. These results indicate that both GS and GlnR are required for the repression of nitrogen fixation under nitrogen-excess condition.

qRT-PCR analysis showed that the transcription levels of *nifH* gene were similar in both Δ*glnA1* and wild-type strains under both nitrogen-limited and -excess conditions, in agreement with nitrogenase activity in these strains and suggesting that GS encoded by *glnA1* is not involved in regulation of *nif* gene expression (Fig 2B). In contrast, the *nifH* gene in Δ*glnA* mutant was transcribed constitutively at modest level under both nitrogen-limited and -excess conditions, in agreement with nitrogenase activity in Δ*glnA* mutant. Transcription levels of the *nifH* gene in Δ*glnRA* double mutant were at basal low level under both nitrogen-limited and -excess conditions, in agreement with nitrogenase activity in this strain. These data suggest that GlnR and GS encoded by *glnA* within *glnRA* operon are responsible for negative regulation of *nif* gene expression according to nitrogen availability.

### C-terminal deletion of GlnR, purification and interaction of GlnR with GS

GlnR protein of *B*. *subtilis* has a high sequence similarity at the N terminus with TnrA, but the C-terminal signal transduction domain of GlnR is sequentially distinct from TnrA and contains an extra 15 residues [20,23,24].

Here, sequence alignments showed that the 137-residue GlnR protein of *P*. *polymyxa* WLY78 exhibited 54% and 40% identity with GlnR and TnrA of *B*. *subtilis* 168, respectively (S3 Fig). GlnR, GS and GS1 from *P*. *polymyxa* WLY78 with His_6_-tag at the N-terminus were overexpressed and purified in *Escherichia coli*, respectively. Also, GlnR^Δ25^, a truncated GlnR with a deletion of the last 25 C-terminal codons (aa 113–137) was overexpressed and purified in *E*. *coli*. Of these purified proteins, GlnR was further evaluated by size-exclusion chromatography analysis. The GlnR protein was eluted as a broad peak with two maxima. Judged from the elution positions of marker proteins, this profile could reflect the coexistence of His_6_-GlnR monomers and dimers in non-instantaneous equilibrium (sequence-deduced masses of His_6_-GlnR monomers and dimers are 19.6 kDa and 39.2 kDa a respectively) (S4A Fig). SDS-PAGE revealed for the two maxima the same band with the expected mass for His_6_-GlnR (S4B Fig). Our results are different from some reports that *P*. *riograndensis* SBR5 GlnR is mainly the dimeric form [14] and *B*. *subtilis* GlnR is mainly monomeric form [20].

**Fig 3:**
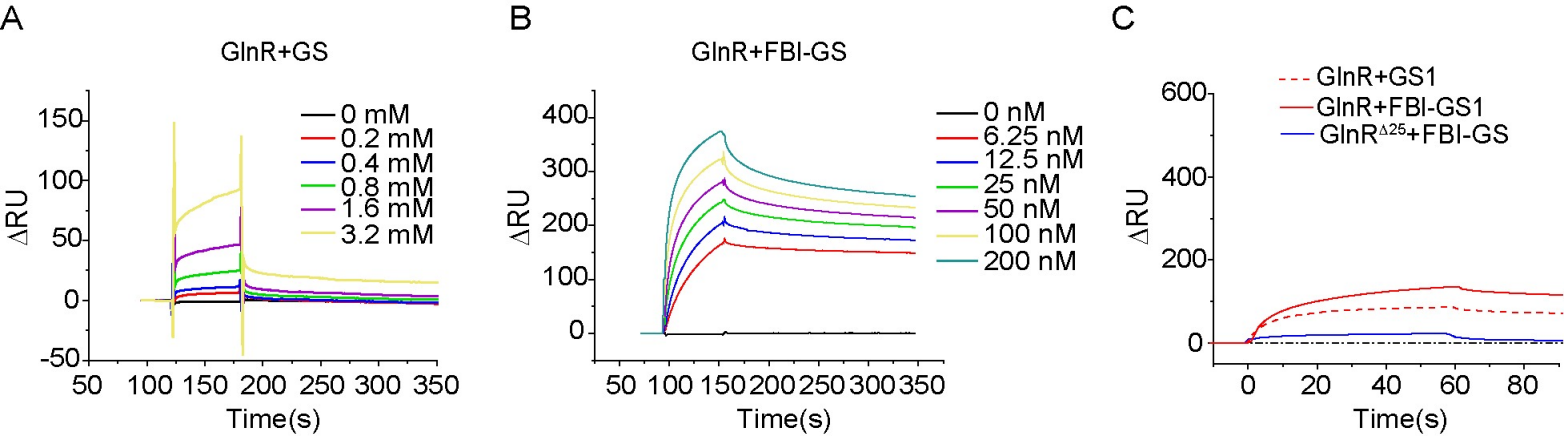
SPR analysis of interaction of GlnR with GS proteins. **A.** Interaction between GS and GlnR. **B.** Interaction between FBI-GS (GS and 1 mM glutamine) and GlnR. **C.** Interactions between GS1 and GlnR (red dashed line), FBI-GS1 (GS1 and 1 mM glutamine) and GlnR (red continuous line), and interaction between FBI-GS and GlnR^Δ25^ (blue continuous line). 100 nM GS1 alone or with 1 mM glutamine was injected onto the chip surface-immobilized GlnR. For the interaction between FBI-GS and GlnR^Δ25^, 100 nM GS with 1 mM glutamine was injected onto the chip surface-immobilized GlnR^Δ25^.

Then, the interaction of GlnR with GS proteins was evaluated by surface plasmon resonance (SPR) assay. His_6_-tagged GlnR was immobilized on a Ni-nitrilotriacetic acid-activated chip sensor surface. Then different concentrations of GS and FBI-GS (GS and glutamine) were loaded onto the GlnR chip surface. In the absence of glutamine, only a weak interaction between GlnR and GS was observed, and the GlnR-GS complex also dissociated quickly even when the concentration of GS was increased from 200 nM to 3.2 mM (Fig 3A). In contrast, in the presence of glutamine, there was still strong interaction between GlnR and FBI-GS even when the concentration of GS protein was decreased from 200 nM to 6.25 nM (Fig 3B). These results indicated that GlnR of *P*. *polymyxa* WLY78 interacted with the feedback inhibited GS form (FBI-GS). Our results are consistent with the reports that GS, in its feedback inhibited form, interacts with GlnR of *P*. *riograndensis* SBR5 [14] and GlnR and TnrA of *B*. *subtilis* [18,20,21]. However, nearly no interaction between GlnR^Δ25^ and FBI-GS was observed (Fig 3C), suggesting that the C-terminal domain is required for interaction between GlnR and FBI-GS.

In contrast, only a basal weak interaction between GlnR and GS1 was detected whether the feedback inhibitor glutamine was present or not (Fig 3C), in agreement with the above-described results that mutation of *glnA1* did not affect regulation of *nif* transcription and nitrogenase activity.

### FBI-GS enhances the in vitro and in vivo DNA-binding activity of GlnR and the C-terminal domain of GlnR affects DNA-binding

**Fig 4:**
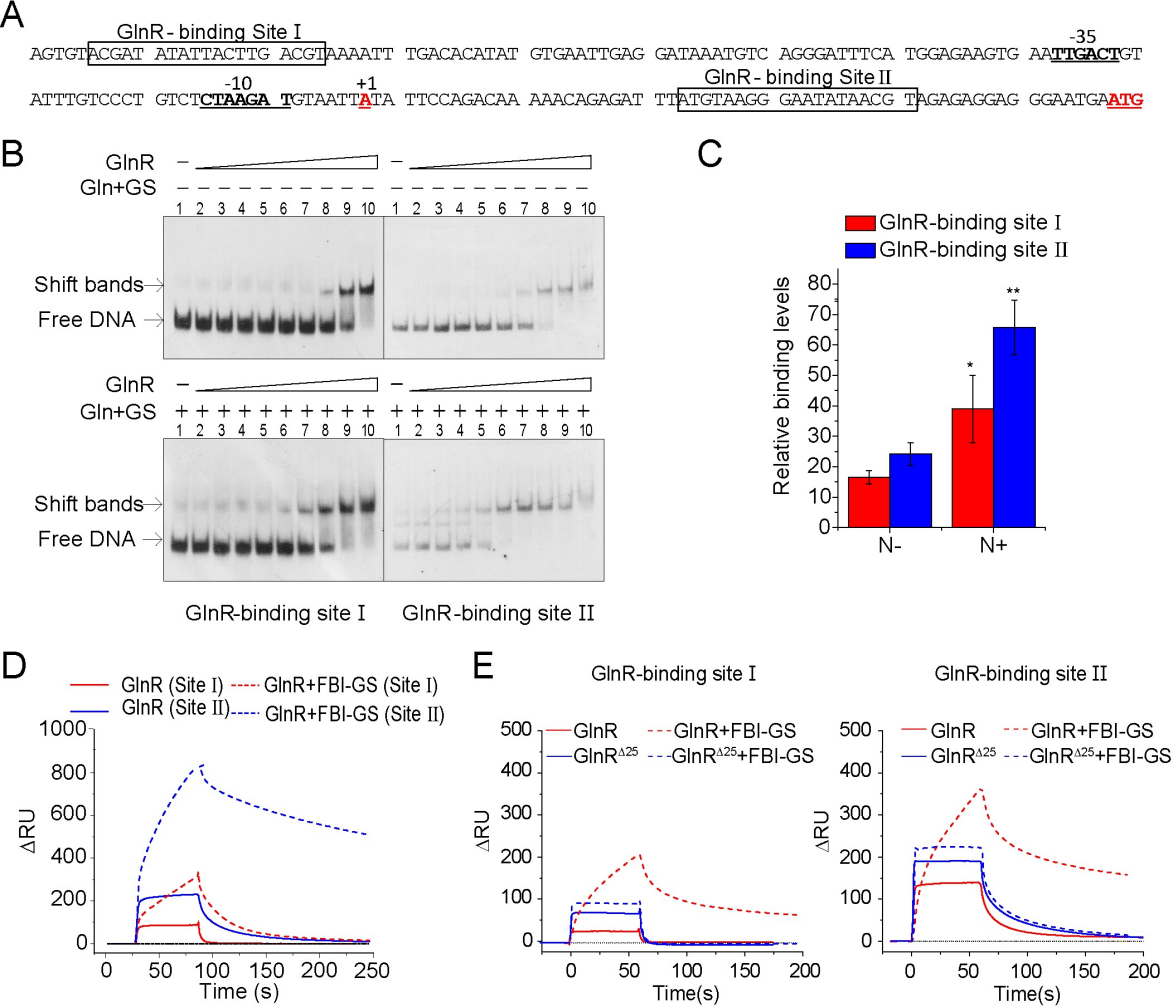
The in vitro and in vivo binding of GlnR protein to two GlnR-binding sites in the *nif* promoter region of *P*. *polymyxa* WLY78. **A.** Prediction of two GlnR-binding sites in the *nif* promoter region. **B.** EMSA revealed the in vitro binding of GlnR to the two GlnR-binding sites. Two DNA fragments: a 59 bp DNA fragment harboring the GlnR-binding site Ⅰ and a 53 bp DNA fragment carrying GlnR-binding site Ⅱ, were synthesized and biotin-labeled. The biotin-labeled DNA fragments were incubated with His-GlnR supplemented without or with FBI-GS (5 mM glutamine and 500 nM His-GS). Lane 1 contained no GlnR. Lanes 2–10 contained increasing concentrations of His-tagged GlnR (4, 8, 16, 32, 64, 128, 256, 512, 1024 nM). **C.** ChIP-qPCR assays revealed in vivo biding of GlnR to both GlnR-binding sites under both nitrogen-limited (N-) and -excess (N+) conditions. The binding levels of control (Δ*glnR*) were arbitrarily set to 1.0. Error bars indicate SD from three independent experiments. **P < 0.01; *P < 0.05. **D.** SPR analysis of GlnR binding to both GlnR-binding sites. 500 nM GlnR alone or with FBI-GS (GS + 1 mM glutamine) was injected onto the chip surface-immobilized DNA fragments harboring GlnR-binding site Ⅰ or site Ⅱ. GlnR (Site Ⅰ) indicates the binding of GlnR alone to site Ⅰ; GlnR (Site Ⅱ) indicates the binding of GlnR alone to site Ⅱ; GlnR+FBI-GS (Site Ⅰ) indicates the binding of GlnR plus FBI-GS to site Ⅰ; GlnR+FBI-GS (Site Ⅱ) indicates the binding of GlnR plus FBI-GS to site Ⅱ. **E.** SPR analysis of the binding affinity of the truncated GlnR (GlnR^Δ25^) protein to the two GlnR-binding sites. 500 nM GlnR or GlnR^Δ25^ alone or with FBI-GS (GS + 1 mM glutamine) was injected onto the chip surface-immobilized DNA fragments harboring GlnR-binding site Ⅰ or site Ⅱ. In comparison with wild-type GlnR, GlnR^Δ25^ protein has the increased binding affinity to both sites, especially site Ⅱ. The addition of FBI-GS does not obviously increase the binding affinity of GlnR.

We predicted that the promoter region of *nif* operon of *P*. *polymyxa* WLY78 contained two GlnR-binding sites: GlnR-binding site Ⅰ and GlnR-binding site Ⅱ (Fig 4A and S5 Fig) by using MEME/MAST software [31]. The two sites were 118 bp separate. Site Ⅰ was located 58 bp upstream of -35 regions of *nif* promoter, and site Ⅱ was seated 24 bp downstream of the *nifB* transcription start site. The binding motifs of the two sites resembled the common consensus sequences (5’-TGTNAN7TNACA-3’) of the GlnR/TnrA-binding site [15,32,33].

To determine whether the two GlnR-binding sites are direct targets of GlnR, the in vitro and in vivo binding of GlnR protein to the two GlnR-binding sites were performed by using electrophoretic mobility shift assays (EMSA), surface plasmon resonance (SPR) spectroscopy and chromatin immunoprecipitation-quantitative PCR (ChIP-qPCR). EMSA experiments revealed that in vitro GlnR bound to the two sites (Fig 4B). Addition of both GS and glutamine (the feedback-inhibited GS) enhanced the binding affinity of GlnR to the two sites (Fig 4B). Also, the addition of both GS and glutamine did not change the band positions of the DNA-GlnR protein complex, supporting that FBI-GS did not directly bind to DNA and it functioned as a chaperon to activate the DNA-binding activity of GlnR [20,21].

Then, ChIP-qPCR experiments were performed to investigate the in vivo binding of GlnR to the two GlnR-binding sites. GlnR polyclonal antibody was used to measure binding of GlnR to its target and qRT-PCR with primers corresponding to the GlnR-binding site Ⅰ and site Ⅱ was performed. As shown in Fig 4C, GlnR bound to the both sites under both nitrogen limitation and nitrogen excess conditions, but the binding levels of GlnR to both sites were much higher under excess nitrogen than under nitrogen limitation. These findings agree with the results obtained by EMSA. Also, Fig 4C shows that the binding level of GlnR to site Ⅱ was higher than to site Ⅰ under both conditions.

Furthermore, we tested the in vitro affinity of GlnR to the two GlnR-binding sites by SPR spectroscopy. This SPR assay demonstrated that GlnR alone could specifically bind to the two GlnR-binding sites, but the affinity of GlnR for site Ⅱ was much stronger than for site Ⅰ. Regardless of the interaction intensity with each site, in the absence of glutamine, GlnR-DNA binding was transient and unstable due to quick dissociation (Fig 4D). However, addition of both GS and glutamine (the feedback-inhibited GS) greatly stabilized the DNA-protein complex. Especially, FBI-GS significantly stabilized the DNA (site Ⅱ)-protein complex, consistent with the classical function of GlnR as a repressor [15,16].

The affinity of the truncated GlnR (GlnR^Δ25^) protein to the two GlnR-binding sites was also investigated by SPR assay. As shown in Fig 4E, in comparison with wild-type GlnR, GlnR^Δ25^ protein had higher affinity for both sites. The addition of FBI-GS greatly stabilized the DNA-GlnR complex, but it did not have obvious effect on the DNA-GlnR^Δ25^ complex. Our results indicate that the C-terminal region of *P*. *polymyxa* GlnR is an autoinhibitory domain that inhibits DNA-binding ability of GlnR and that the C-terminal domain is also required for the interaction between FBI-GS and GlnR, consistent with the observations in *B*. *subtilis* GlnR [20,21].

**Fig 5:**
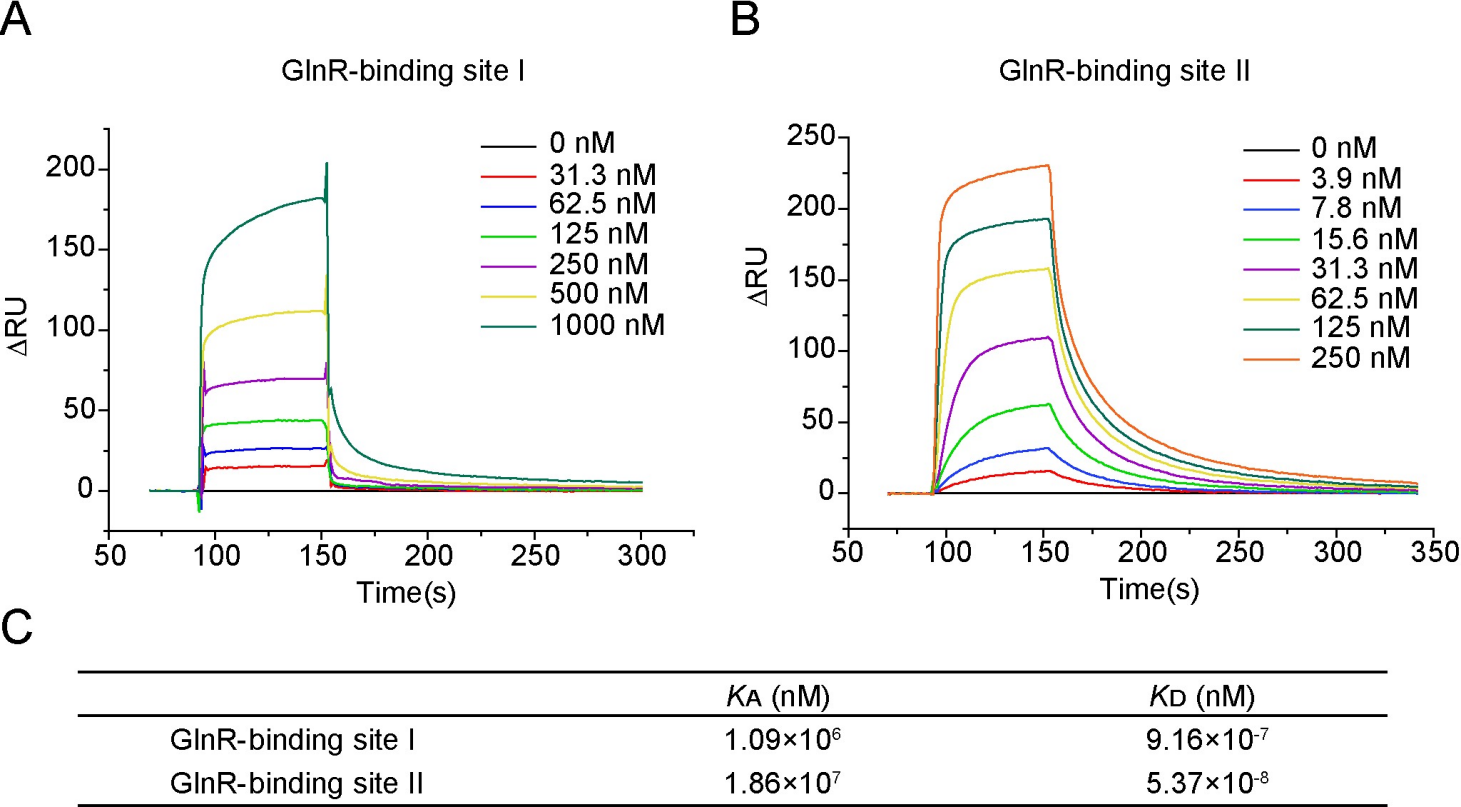
Binding affinity of GlnR to GlnR-binding site Ⅰ and GlnR-binding site Ⅱ. **A.** SPR titration analysis of GlnR binding to GlnR-binding site Ⅰ. **B.** SPR titration analysis of GlnR binding to GlnR-binding site Ⅱ. **C.** Binding affinity of GlnR to GlnR-binding site Ⅰ and GlnR-binding site Ⅱ.

To clarify the affinity of GlnR to both sites, quantitative evaluation was carried out with SPR. A double-stranded DNA oligomer that contained the sequence of site Ⅰ or site Ⅱ was fixed onto the chip as described in Material and Methods. Different concentrations of GlnR protein were loaded onto the DNA chip surface. As shown in Fig 5A and 5B, there was no binding signal in the absence of GlnR and the binding signals became strong with the increase of concentrations of GlnR protein. When the concentration of GlnR was increased to 500 nm and 1000 nM, an obvious binding of GlnR to site Ⅰ was found, but it dissociated quickly, indicating that the binding of GlnR to site Ⅰ is transient and unstable. In contrast, the binding of GlnR to site Ⅱ was stronger and it dissociated slowly, indicating that the binding of GlnR to site Ⅱ is stronger than to site Ⅰ due to slow dissociation. The corresponding *K*_A_ and *K*_D_ values for site Ⅰ were calculated to be 1.09×10^6^ and 9.16×10^−7^, respectively. Whereas the *K*_A_ and *K*_D_ values for site Ⅱ were 1.86×10^7^ and 5.37×10^−8^, respectively (Fig 5C). The values of *K*_A_ for site Ⅱ was consistently higher than that for site Ⅰ, and the values of *K*_D_ for site Ⅱ was much lower than that for site Ⅰ, indicating that affinity of site Ⅱ for GlnR is higher than site Ⅰ. Our current results are different from those obtained in *P*. *riograndensis* SBR5 where GlnR bound to the two GlnR-binding sites [PnifM(1) and PnifM(2)] of the main *nif* gene cluster at similar levels and whose GlnR affinity for site Ⅰ was slightly higher than for site Ⅱ [14].

### GlnR-binding site Ⅰ and Site Ⅱ are involved in positively and negatively regulating *nif* gene transcription, respectively

**Fig 6:**
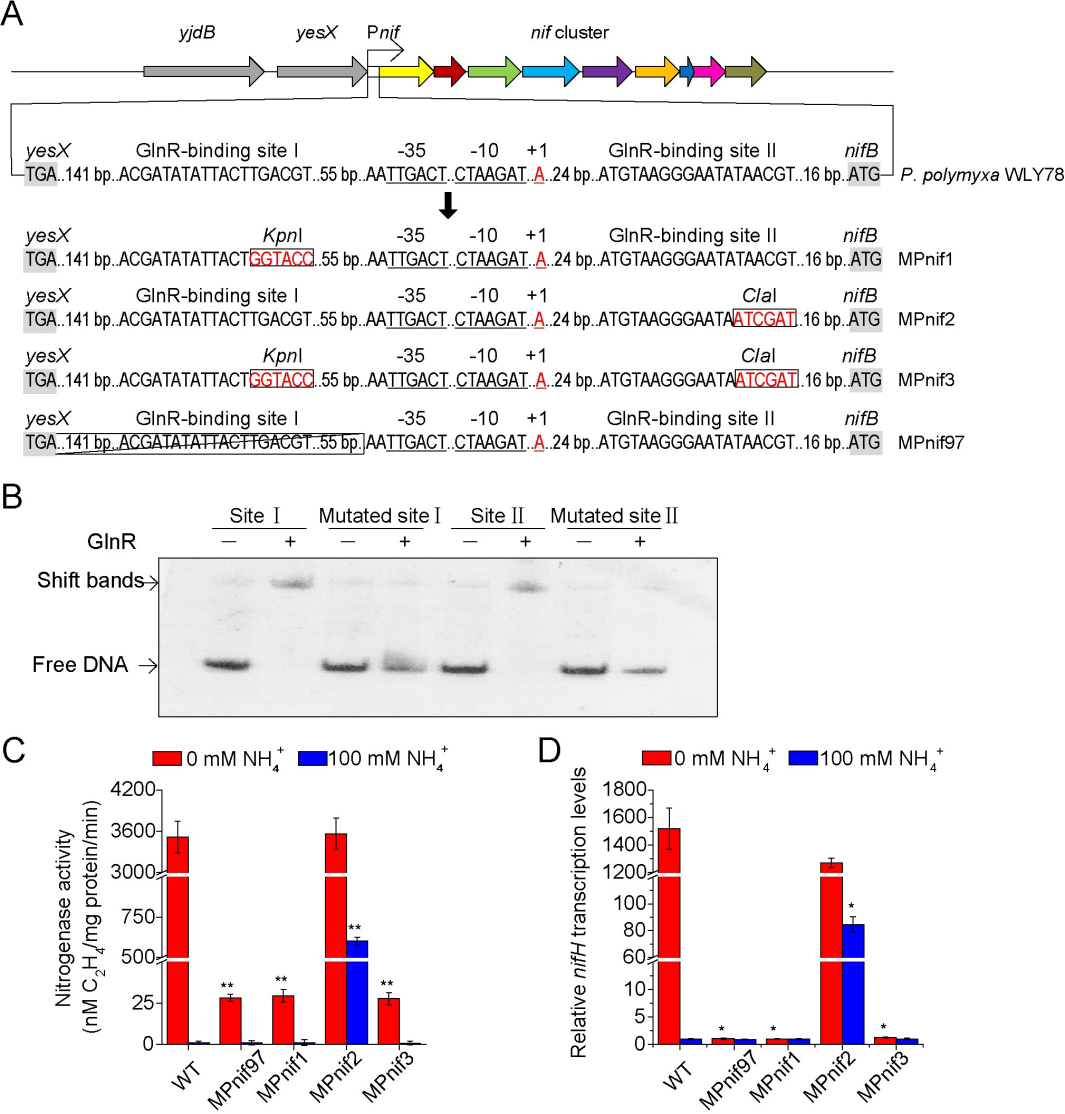
Mutation analysis of the GlnR-binding sites in *P*. *polymyxa* WLY78. **A.** A schematic site-specific mutation or deletion of the GlnR-binding sites. **B.** EMSA verification of the binding affinity of GlnR to the mutated GlnR-biding site Ⅰ and GlnR-binding site Ⅱ. Site Ⅰ indicates wild-type GlnR-binding site Ⅰ; Site Ⅱ indicates wild-type GlnR-binding site Ⅱ.–and + indicate that the absence and presence of GlnR protein, respectively. **C.** Nitrogenase activity in wild-type (WT) and four mutants MPnif1, MPnif2, MPnif3 and MPnif97 under both nitrogen-limited and -excess conditions. **D.** qPCR analysis of the relative transcription levels of the *nifHDK* genes in the wild-type and mutant strains under both nitrogen-limited and -excess conditions. The transcription levels of *nifH* in the WT strain under nitrogen-excess condition were arbitrarily set to 1.0. Results are representative of at least three independent experiments. Error bars indicate SD. **P < 0.01; *P < 0.05.

Since GlnR was positively and negatively involved in the regulation of nitrogen fixation according to nitrogen availability, both increase and decrease of nitrogenase activity could be expected through mutations of the two GlnR-binding sites in the *nif* promoter region. Thus, the site-specific mutagenesis of the two GlnR-binding sites was performed. As shown in Fig 6A, the consensus sequence TGACGT in site Ⅰ region was replaced with a restriction site of *Kpn* Ⅰ (GGTACC) via homologous recombination, generating mutant MPnif1. The consensus motif ATAACG in site Ⅱ was replaced by a restriction site of *Cla* Ⅰ (ATCGAT), which generated the mutant MPnif2. A double mutant MPnif3 with mutations of both GlnR-binding sites was generated. Also, the mutant MPnif97 with deletion of site Ⅰ was also constructed. EMSA confirmed that GlnR did not bind to the mutated sites (Fig 6B).

In comparision with wild-type strain, only basal nitrogenase activity was observed in both mutants MPnif1 and MPnif97 under both nitrogen-limited and -excess conditions (Fig 6C), suggesting that site Ⅰ is essentially required for nitrogen fixation. The data are consistent with nitrogenase activity in Δ*glnR* mutant, indicating that site Ⅰ is the target of GlnR. In contrast, nitrogenase activity in mutant MPnif2 was derepressed partially under nitrogen-excess condition, suggesting that site Ⅱ is involved in repressing nitrogen fixation. The data are consistent with nitrogenase activity in Δ*glnA* mutant, suggesting that site Ⅱ is the target of GS encoded by *glnA*. Nitrogenase activity in the double mutant MPnif3 was nearly abolished under both nitrogen-limited and excess conditions, in agreement with nitrogenase activities in Δ*glnRA* double mutant. The *nif* gene transcription levels determined by qRT-PCR (Fig 6D) were consistent with the nitrogenase activities in mutants MPnif97, MPnif1, MPnif2 and MPnif3.

Taken together, these results indicate that GlnR binds to site Ⅰ to activate *nif* expression under nitrogen-limited condition and binds to site Ⅱ to repress *nif* transcription under nitrogen-excess condition. FBI-GS is involved in repressing *nif* transcription by its interaction with GlnR under excess nitrogen.

## Discussion

GlnR is a global transcription regulator of nitrogen metabolisms found extensively in *Bacillus* and other Gram-positive bacteria. It generally acts as a repressor repressing the transcription of *glnRA* operon, *tnrA* and *ureABC* in *B*. *subtilis* under excess nitrogen [15,16,25]. TnrA is another transcription regulator of nitrogen metabolisms found mainly in *Bacillus* and it serves in most cases as an activator under nitrogen limitation [26]. In the present work, we reveal that *P*. *polymyxa* GlnR simultaneously acts as an activator and a repressor for nitrogen fixation by binding to different loci of the single *nif* promoter region according to nitrogen availability. GS is ne cessarily required for *nif* repression mediated by GlnR.

In this study, two GlnR-binding sites flanking the -35/-10 regions of the promoter of *nif* operon in *P*. *polymyxa* WLY78 is predicted by software and then confirmed by in vitro EMAS and SPR experiments and by in vivo ChIP-qPCR. The two sites are 118 bp separated. Site Ⅰ is located 58 bp upstream of -35 region of *nif* promoter, and site Ⅱ is seated 24 bp downstream of the *nifB* transcription start site. The location of site Ⅰ is an indicative of activation site, since regulator, such as TnrA, bound at this position most likely activates gene transcription [23,25,26]. Site Ⅱ located just downstream of promoter is an indicative of repression site, since regulator bound at this site will represses gene transcription by sterically hindering RNA extension [34]. The binding motif (5’-TGTAAGGGAATATAACG-3’) of site Ⅱ possesses the common consensus sequence (5’-TGTNAN7TNACA-3’) of the GlnR-binding site (S5 Fig), while the consensus sequences (5’-CGATATATTACTTGACG-3’) of site Ⅰ fit the TnrA-specific motif (5’-NGNNAN7TNACN-3’) which clearly lacks the conserved A and T at the 3′ and 5′ end [15,32,33]. Our studies of deletion or mutagenesis of GlnR-binding site Ⅰ, site Ⅱ and both sites demonstrated that site Ⅰ is responsible for activating *nif* expression and site Ⅱ is required for repressing *nif* transcription, in agreement with the locations of the two sites. Two GlnR-binding sites flanking the -35/-10 *nif* promoter region were also found in *P*. *riograndensis* SBR5 [14], but they exhibit a little difference with those of *P*. *polymyxa* WLY78 in the precise consensus sequences and locations. As shown in S6 Fig, the site Ⅱ in *P*. *polymyxa* WLY78 is located 16 bp upstream of ATG (translation start site), while the O_A_-*nifB* (site Ⅱ) in *P*. *riograndensis* SBR5 is located 60 bp upstream of ATG. A common consensus sequence (TGTNAN7TNACA) of GlnR-binding motif in the class *Bacillus* (S5 Fig) is more conserved in the two GlnR-binding sites of *P*. *riograndensis* SBR5 than in those of *P*. *polymyxa* WLY78. SPR assay demonstrated that GlnR-binding site Ⅰ of *P*. *riograndensis* SBR5 displayed higher affinity for GlnR, whereas the second site had lower affinity and dissociated faster [14]. In contrast, GlnR-binding site Ⅰ of *P*. *polymyxa* WLY78 exhibited lower affinity for GlnR and dissociated faster, while site Ⅱ displayed higher affinity due to slow dissociation, especially in the presence of FBI-GS.

Based on the two binding sites in the *nif* promoter region of *P*. *riograndensis* SBR5, a DNA-looping model that represents a strong and strict regulation for *nif* genes was proposed [14]. In this model, DNA loop formation was induced by two GlnR dimers bound to both GlnR-binding sites and bridged by feedback-inhibited GS. However, our results from deletion and complementation analyses of *glnR*, *glnA* and *glnRA* and from mutation analyses of GlnR-binding sites did not support the DNA-looping model. Our data demonstrate evidently that GlnR bound to site Ⅰ in a weak and transient way and then activated *nif* gene transcription under nitrogen limitation, and the FBI-GS stabilized the binding affinity of GlnR to binding site Ⅱ and the strong binding of GlnR to site Ⅱ repressed *nif* gene transcription by interfering RNA extension under nitrogen-excess condition. Our studies that mutation of the 4–5 nucleotides in the half-sequences within the GlnR-binding site Ⅰ (ACGATATATTACTTGACGT) or site Ⅱ (ATGTAAGGGAATATAACGT) resulted to no binding of GlnR are consistent with that 4 nucleotides in each operator half-site of the DNA consensus sequence (TGTNAN7TNACA) were required for GlnR/TnrA specific DNA binding [18,23,26,35]. Our data also support that TnrA/GlnR form a weak symmetric dimer by binding their palindromic cognate sites [18,21]. Thus we think that it is unlikely for GlnR to be a tetramer formed by interacting between two dimers bound on the two GlnR-binding sites. There are also two GlnR-binding sites in the promoter region of *B*. *subtilis glnRA*, one of which lies immediately upstream of the -35 promoter element and the other site overlaps the -35 region [36]. It was previously reported that GlnR bond to these sites in a cooperative manner, and both sites were required for full repression of *B*. *subtilis glnRA* [37].

Mutation of GlnR-binding site Ⅱ made the mutant MPnif2 have nitrogenase activities and express *nif* genes under both nitrogen-limited and -excess conditions (Fig 6C and 6D), supporting that GlnR bound to site Ⅰ to activate *nif* gene transcription under nitrogen limitation. However, the nitrogenase activity and *nif* gene transcription in mutant MPnif2 did not reach similar levels under both nitrogen-limited and -excess conditions. We deduce that perhaps the mutation of site Ⅱ made the mutant MPnif2 have more FBI-GS proteins to strength the binding of GlnR to site Ⅰ and then interfere *nif* gene transcription under nitrogen excess. However, under normal physiological condition, since there are two GlnR-binding sites and the affinity of GlnR for site Ⅱ was much stronger than for site Ⅰ, repression of *nif* gene transcription was mediated by site Ⅱ. Whether site Ⅰ, together with site Ⅱ was involved in repressing *nif* gene transcription under nitrogen-rich condition needs to be determined in the future.

Our study by deletion, complementation and overexpression of *glnR*, *glnA* and *glnRA* and by mutagenesis or deletion of GlnR-binding sites reveals that GlnR bound to GlnR-binding site Ⅰ and activated *nif* transcription under nitrogen limitation, and GlnR bound to GlnR-binding site Ⅱ and repressed *nif* transcription under excess nitrogen. The novel, dual positive and negative regulatory mechanism is for the first time reported in nitrogen fixation. Although dual function of GlnR in *Streptomyces hygroscopicus* var. *jinggangensis* 5008 was reported [38], *Streptomyces* GlnR is an OmpR-like response regulator which does not display any similarity to the *Paenibacillus*/*Bacillus* GlnR regulator belonging to the MerR family [39].

Although GlnR protein alone could bind to the two sites in *nif* promoter of *P*. *polymyxa* WLY78, this binding was transient and unstable. We deduce that the transient GlnR-DNA interaction is sufficient for GlnR to act as an active regulator. Interestingly, under nitrogen-limited condition, TnrA (but not GlnR) of *B*. *subtilis* is further stabilized by an interaction with GlnK [40,41]. It also reported that GlnR protein exhibited an increased affinity for the *glnRA* operon promoter when bound to GlnK in *Streptococcus mutans* [42]. Whether GlnR dimer is stabilized by GlnK in *P*. *polymyxa* WLY78 under nitrogen-limited condition needs to be investigated in the future.

It is well characterized that the C-terminal domain of *B*. *subtilis* GlnR protein is sequentially distinct from TnrA and contains an extra 15 residues [23]. This region acts as an autoinhibitory domain that prevents GlnR dimerization and thus inhibits DNA binding [20–22]. FBI-GS acts as a chaperone to stabilize dimerization and subsequent DNA binding of GlnR [20–22]. In this study, the *P*. *polymyxa* GlnR^Δ25^, a truncated GlnR with a deletion of the last 25 C-terminal codons was overexpressed and purified in *E*. *coli*. SPR analyses show that the interaction between GlnR^Δ25^ and GS is greatly decreased compared to wild-type GlnR. Also, GlnR^Δ25^ had higher binding affinity to both GlnR-binding sites than wild-type GlnR (Fig 4E). The addition of FBI-GS greatly enhanced the DNA-binding affinity of wild-type GlnR, but it did not obviously increase the DNA-binding affinity of GlnR^Δ25^ protein. FBI-GS also stabilized the DNA-GlnR complex, but it had no effect on the DNA-GlnR^Δ25^ complex. These results reveal that the C-terminal region of *P*. *polymyxa* GlnR is an autoinhibitory domain and it is also involved in the interaction between FBI-GS and GlnR, in agreement with the results obtained in *B*. *subtilis* GlnR. Our results demonstrate that FBI-GS stabilizes the binding of GlnR to the two site, especially site Ⅱ, consistent with the observations in *B*. *subtilis* GlnR [20,21]. We deduce that the monomers in the mixture of dimers and monomers of *P*. *polymyxa* GlnR protein were shifted to dimers by the interaction of FBI-GS with GlnR under excess nitrogen. Consequently, the strong binding of GlnR to site Ⅱ led to the represstion of *nif* transcription by interfering RNA extension. This mode of repressing *nif* gene transcription of *P*. *polymyxa* during excess nitrogen is a classical function of GlnR regulator found extensively in *B*. *subtilis* and some other Gram-positive bacteria. Although the activity of TnrA is also controlled by FBI-GS, the mechanisms of regulation are different between TnrA and GlnR. Under excess nitrogen, FBI-GS forms a stable complex with TnrA, which inhibits its DNA-binding activity [20,24].

TnrA and GlnR are generally recognized as the members of the MerR family regulators according to their common winged-HTH (helix-turn-helix) domains. However, TnrA and GlnR may regulate transcription using molecular mechanisms distinct from MerR proteins. MerR proteins activate transcription by distorting and realigning DNA promoters with nonoptimal spacing between the -10 and -35 boxes [43]. Unlike MerR members, the promoters bound by TnrA and GlnR are optimally arranged and a 17-bp inverted repeat sequences with the consensus TGTNAN7TNACA constitutes the minimal binding site for these proteins [15,32]. It was previously suggested that TnrA functions primarily as an activator by binding operator DNA sites and recruiting RNA polymerase (RNAP) [24,26], whereas GlnR does not bind RNAP and hence functions as a repressor. The recent study on structures has revealed that GlnR induces bend and conformational changes in the DNA similar to those in TnrA [18], supporting our results that GlnR functions as an activator just as TnrA does under nitrogen limitation.

The wild-type *P*. *polymyxa* WLY78 has the highest nitrogenase activity in the absence of NH_4_^+^ and has no activity in the presence of more than 5 mM NH_4_^+^ (Fig 1A). Deletion of *glnR* leads to loss of both nitrogenase activity and *nif* gene transcription under nitrogen limitation, suggesting that GlnR is essential required for activating *nif* gene expression. Deletion of *glnA* makes the Δ*glnA* mutant have both nitrogenase activity and *nif* gene transcription under both nitrogen-limited and -excess conditions, suggesting that GS encoded by *glnA* is involved in repressing *nif* gene transcription under nitrogen-excess condition. Mutation of GlnR-binding site Ⅰ results to loss of both nitrogenase activity and *nif* transcription under nitrogen limitation, suggesting that site Ⅰ is responsible for activating *nif* gene transcription. However, mutation of GlnR-binding site Ⅱ makes the mutant MPnif2 have both nitrogenase activities and *nif* gene transcriptions under both condition, consistent with the nitrogenase and *nif* gene ranscription in Δ*glnA* mutant. Our study with SPR also demonstrates that the affinity of GlnR for site Ⅱ is stronger than for site Ⅰ. Under nitrogen-excess condition, glutamine is synthesized and it feedbacks the GS, yielding FBI-GS, EMSA, SPR and Chip-PCR reveal that the presence of FBI-GS (GS and glutamine) greatly stabilizes the GlnR-DNA complex and decreases the dissociation of GlnR from binding site Ⅱ.

**Fig 7:**
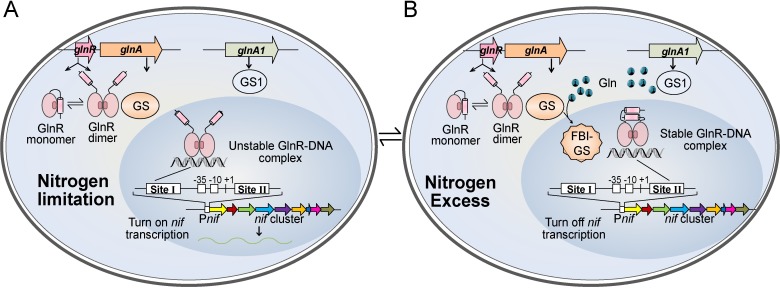
Regulatory model of GlnR involved in nitrogen fixation in *P*. *polymyxa* WLY78. **A.** GlnR protein exists as a mixture of dimer and monomer. Monomer of GlnR is an autoinhibitory form whose C-terminal region folds back and inhibits dimer formation. During nitrogen limitation, dimer of GlnR binds to GlnR-binding site Ⅰ in a weak and transient association way and activates *nif* transcription. **B.** During excess nitrogen, glutamine (Gln) is produced by GS and GS1 catalyzing glutamate and NH_4_^+^. Gln binds to and feedback inhibits GS by forming the complex FBI-GS. FBI-GS interacts with the C-terminal tail of GlnR and relieves its autoinhibition, shifting the monomer to the DNA-binding active form. The FBI-GS further stabilizes the binding affinity of GlnR to GlnR-binding site Ⅱ and thus represses *nif* transcription.

According to our results, we proposed a regulatory model of GlnR involved in nitrogen fixation in *P*. *polymyxa* WLY78 (Fig 7). GlnR exists in a mixture of dimers and monomers. Monomer of GlnR is an autoinhibitory form whose C-terminal region folds back and inhibits dimer formation. Under nitrogen-limited condition, GlnR dimer binds to site Ⅰ in a weak and transient association way and then activates *nif* expression (Fig 7A). Although GlnR also sequentially or simultaneously binds to site Ⅱ, binding of GlnR to this site does not repress *nif* transcription due to GlnR having only a weak and transient association with DNA during this condition. Also, the large amounts of GlnR produced under this condition enable *nif* transcription to carry on, since expression of *glnR* itself is nitrogen-dependent. Under nitrogen-excess condition (Fig 7B), glutamine is in excess and it binds to and feedback inhibits GS by forming the complex FBI-GS. The FBI-GS interacts with the C-terminal tail of GlnR and relieves autoinhibition, shifting the monomer to the DNA-binding active form. The FBI-GS further stabilizes the binding affinity of GlnR to both sites, especially site Ⅱ. The stable binding of GlnR to site Ⅱ blocks the RNA extension and thus represses *nif* transcription.

In conclusion, our combined data reveal a novel molecular regulatory mechanism of nitrogen fixation in *P*. *polymyxa* WLY78. GlnR binds to site Ⅰ to activate *nif* gene transcription under nitrogen-limited condition, and it binds to site Ⅱ to repress *nif* gene transcription under nitrogen-excess condition. The activity of GlnR is controlled by GS in response to nitrogen availability.

## Materials and methods

### Strains, plasmids and growth conditions

Bacterial strains and plasmids used in this study are summarized in S1 Table. *P*. *polymyxa* strains were grown in nitrogen-limited medium (2 mM glutamate) or nitrogen-excess medium (2 mM glutamate +100 mM NH_4_^+^) under anaerobic condition [12]. For assays of nitrogenase activity, β-galactosidase assays and *nif* expression, *P*. *polymyxa* strains were grown in nitrogen-limited medium or nitrogen-excess medium under anaerobic condition. *Escherichia coli* strains JM109 and BL21 (DE3) were used as routine cloning and protein expression hosts, respectively. Thermo-sensitive vector pRN5101 [44] was used for gene disruption in *P*. *polymyxa*. Shuttle vector pHY300PLK was used for complementation experiment and transcriptional fusion construction. pET-28b(+) (Novagen) was used for expressing recombinant His6-tagged protein in *E*. *coli*. When appropriate, antibiotics were added in the following concentrations: 100 μg/ml ampicillin, 25 μg/ml chloramphenicol, 12.5 μg/ml tetracycline, 50 μg/ml kanamycin, and 5μg/ml erythromycin for maintenance of plasmids.

### Construction of Δ*glnR*, Δ*glnA1*, Δ*glnA* and Δ*glnRA* mutants and their complementation and overexpression strains

The four in-frame-deletion mutants: Δ*glnR*, Δ*glnA1*, Δ*glnA* and Δ*glnRA*, were constructed by a homologous recombination method. The upstream (ca. 1 kb) and downstream fragments (ca. 0.5 kb) flanking the coding region of *glnR*, *glnA1*, *glnA* and *glnRA* were PCR amplified from the genomic DNA of *P*. *polymyxa* WLY78, respectively. The primers used for these PCR amplifications were listed in S2 Table. The two fragments flanking each coding region of *glnR*, *glnA1*, *glnA* and *glnRA* were then fused with *Sal*Ⅰ/*Bam*HⅠ digested pRN5101 vector using Gibson assembly master mix (New England Biolabs), generating the four recombinant plasmids pRDglnR, pRDglnA1, pRDglnA and pRDglnRA, respectively. Then, each of these recombinant plasmids was transformed into *P*. *polymyxa* WLY78 as described by [45], and the single-crossover transformants were selected for erythromycin resistance (Em^r^). Subsequently, marker-free deletion mutants (the double-crossover transformants) Δ*glnR*, Δ*glnA1*, Δ*glnA* and Δ*glnRA* were selected from the initial Em^r^ transformants after several rounds of nonselective growth at 39°C and confirmed by PCR amplification and sequencing analysis.

Complementation for Δ*glnR*, Δ*glnA* and Δ*glnRA* was performed. For complementation of Δ*glnR*, the *glnR* gene and its promoter was inserted into the *amyE* site on genome of Δ*glnR* strain. To do this, two fragments: an 1161 bp DNA fragment and an 1017 bp fragment flanking the *amyE* gene, were PCR amplified from the genomic DNA of *P*. *polymyxa* WLY78, respectively. An 803 bp DNA fragment carrying the *glnR* ORF (414 bp) and its own promoter (389 bp) was also PCR amplified. Then, three fragments and the vector pRN5101 digested with *Bam*HⅠ and *Hind*Ⅲ were fused together using Gibson assembly master mix, generating the recombinant plasmid pRCglnR. The recombinant plasmid pRCglnR was transformed into the cells of Δ*glnR* strain and then double-crossover transformants were selected after several rounds of growth at 39°C. Finally, the complementation strain CglnR which contains an 803 bp DNA fragment carrying *glnR* ORF and its promoter integrated on the *amyE* site was obtained and confirmed by PCR and DNA sequencing. For complementation of Δ*glnRA* mutant, a 2116 bp DNA fragment containing the complete *glnRA* operon and its own promoter was PCR amplified from the genomic DNA of *P*. *polymyxa* WLY78. For complementation of Δ*glnA*, a 1419 bp DNA fragment containing the coding region of *glnA* and a 280 bp promoter region of *glnRA* operon were PCR amplified, respectively, and then the two fragments were fused together using Gibson assembly master mix. These fragments were digested with *BamH*Ⅰ/*Sal*Ⅰ, and ligated into vector pHY300PLK, generating *glnA*-complemented vector pHYglnA and *glnRA*-complemented vector pHYglnRA, respectively. Each of these recombinant plasmids was correspondingly transformed into Δ*glnA* and Δ*glnRA* mutants, and tetracycline-resistant (Tet^r^) transformants were selected and confirmed by PCR and sequencing.

The strain WT/*glnR* in which *glnR* is overexpressed was also constructed. An 803 bp DNA fragment carrying the *glnR* ORF (414 bp) and its own promoter (389 bp) was PCR amplified and then ligated to multicopy vector pHY300PLK and then transformed to *P*. *polymyxa* WLY78, generating the *glnR* overexpression strain. The primers used here are listed in S2 Table.

### Construction of mutants with deletion or mutagenesis of the GlnR-binding site(s)

Four mutants with deletion or mutagenesis of the GlnR-binding site(s) were performed via homologous recombination. A 313 bp *nif* promoter region (from -253 to +60 relative to the *nifB* transcription start codon) containing both of the GlnR-binding sites Ⅰ and Ⅱ was used as a target for mutation. Thus, three 313-bp DNA fragments PnifM1, PnifM2 and PnifM3 (S3 Table) were synthesized based on the sequences of *nif* promoter region. Notably, PnifM1 contains the mutated GlnR-binding site Ⅰ where the last six base pairs TGACGT within the 19-bp consensus sequences (ACGATATATTACT TGACGT) of the GlnR-binding site Ⅰ were replaced by a restriction site of *Kpn*Ⅰ (GGTACC). PnifM2 carries the mutated GlnR-binding site Ⅱ where the last six base pairs ATAACG within the 19-bp consensus sequences (ATGTAAGGGAAT ATAACG) was replaced by a restriction site of *Cla*Ⅰ (ATCGAT). PnifM3 contains both of mutated site Ⅰ and site Ⅱ where the consensus motifs TGACGT and ATAACG were simultaneously replaced by restriction sites *Kpn*Ⅰ and *Cla*Ⅰ. The three DNA fragments PnifM1, PnifM2 and PnifM3 were then cloned to plasmid pUC19, respectively. Then, each of the three fragments PnifM1, PnifM2 and PnifM3 (S3 Table) was PCR amplified from the recombinant plasmids. Two homologous arms (1205 bp and 1101 bp) flanking the 313 bp region *in nif* promoter were amplified from the genomic DNA of *P*. *polymyxa* WLY78 using the primers MPnif1/MPnif2 and primers MPnif5/MPnif6 (S4 Table), respectively. Each of the two arms contains ca. 20 bp overlap with the above-described 313 bp DNA fragments (PnifM1, PnifM2 and PnifM3). Then, the two arms and the DNA fragments PnifM1, PnifM2 and PnifM3 were assembled to the *Bam*HⅠ/*Hind*Ⅲ digested plasmid vector pRN5101, yielding the recombinant plasmids pRMP1, pRMP2, pRMP3. Each of these recombinant plasmids was introduced into *P*. *polymyxa* WLY78 by transformation. The single-crossover transformants were selected for erythromycin resistance (Em^r^). Subsequently, the double-crossover transformants were selected from the initial Erythromycin resistance transformants after several rounds of nonselective growth at 39°C. These mutants were confirmed by PCR amplification using the primers and subsequent digestion with *Kpn*Ⅰ or *Cla*Ⅰ and then by DNA sequencing. The mutant with deletion of site Ⅰ was also constructed as follows. A 1418 bp DNA upstream fragment and an 1101 bp downstream fragment were PCR amplified from the genomic DNA of *P*. *polymyxa* WLY78. The two fragments were assembled to vector pRN5101, yielding the recombinant plasmid pRMP100 and then the plasmid was transformed into *P*. *polymyxa* WLY78. The mutant with deletion of 213 bp fragment (from -40 bp to -253 bp relative to the *nifB* transcription start codon) containing GlnR-binding site Ⅰ was obtained as described above.

### Construction of a *nif* promoter-*lacZ* fusion (P*nif-lacZ* fusion)

A 313 bp of the native *nif* promoter (P*nif*) (from -253 to +60 relative to the *nifB* transcription start codon) containing both of the GlnR-binding sites Ⅰ and Ⅱ was amplified from the genomic DNA of *P*. *polymyxa* WLY78 using primers LPnif1 and LPnif2 (S5 Table). The *lacZ* coding region was PCR amplified with primers LPnif3 and LPnif4 from the plasmid pPR9TT. The two PCR-amplified fragments were fused together with vector pHY300PLK and then it was transformed into *P*. *polymyxa* WLY78.

### Expression and purification of GlnR and GS proteins in *E*. *coli*

The *glnR*, truncated *glnR* (GlnR^Δ25^, for C-terminal deletion of GlnR, removing the last 25 amino acid residues), *glnA* within *glnRA* operon and *glnA1* were PCR amplified from the genomic DNA of *P*. *polymyxa* WLY78, respectively. These PCR products were cloned into pET-28b(+) (Novagen) to construct tagged proteins with His-tag at the N-terminus and then transformed into *E*. *coli* BL21 (DE3). The recombinant *E*. *coli* strains were cultivated at 37°C in LB broth supplemented with 50 μg/ml kanamycin until mid-log phase, when 0.2 mM IPTG was added and incubation continued at 20°C for 8 hours. Cells were collected and disrupted in a lysis buffer (50 mM NaH_2_PO_4_, 300 mM NaCl, 10 mM Imidazole) by sonication on ice. Recombinant His_6_-tagged proteins in the supernatant were purified on Ni_2_-NTA resin (Qiagen, Germany) according to the manufacturer’s protocol. Fractions eluted with 250 mM imidazole were dialyzed into storage buffer (10 mM Tris-HCl pH7.5, 1 mM EDTA, 80 mM NaCl, 20% (v/v) glycerol) for antibody production or binding buffer (20 mM HEPES pH 7.6, 1 mM EDTA, 10 mM (NH_4_)_2_SO_4_, 1 mM DTT, 0.2% Tween 20, 30 mM KCl) for electrophoretic mobility shift assays (EMSA) and HBS-Mg buffer (10 mM HEPES pH 7.4, 300 mM NaCl, 3 mM MgCl_2_, and 0.005% Nonidet P-40) for surface plasmon resonance spectroscopy (SPR). Purified His-GlnR was used to raise polyclonal rabbit antibody (Beijing Protein Innovation) and for size-exclusion chromatography. Primers used here are listed in S5 Table.

### RNA preparation and qRT-PCR analysis

Transcription levels of genes were compared among *P*. *polymyxa* WLY78 strain and Δ*glnR*, Δ*glnA* and Δ*glnRA* mutants by quantitative real-time RT-PCR (qRT-PCR) analysis. At each experimental time point, 50 ml of culture were harvested and rapidly frozen under liquid nitrogen. Total RNAs were extracted with RNAiso Plus (Takara, Japan) according to the manufacturer’s protocol. Remove of genome DNA and synthesis of cDNA were performed using PrimeScript RT reagent Kit with gDNA Eraser (Takara, Japan). qRT-PCR was performed on Applied Biosystems 7500 Real-Time System (Life Technologies) and detected by the SYBR Green detection system with the following program: 95°C for 15 min, 1 cycle; 95°C for 10 s and 65°C for 30 s, 40 cycles. Primers used for qRT-PCR are listed in S6 Table. The relative expression level was calculated using ΔΔCt method. 16S rRNA was set as internal control and the expression levels of genes in WT strain under nitrogen-excess condition were arbitrarily set to 1.0. Each experiment was performed in triplicate.

### Electrophoretic mobility shift assays (EMSAs)

EMSAs were performed as described previously using a DIG Gel Shift Kit (2nd Generation; Roche, USA) [12]. The promoter fragments of *nif* operon were synthesized by Sangon Biotech Co., Ltd (Shanghai). Two DNA fragments corresponding to the sequences of the first strand and the complementary DNA strand were synthesized. The two strands were annealed and then labeled at the 30 end with digoxigenin (DIG) using terminal transferase, and used as probes in EMSAs. Each binding reaction (20 μl) consisted of 1 μg poly [d(A-T)], 0.3 nM labelled probe, and various concentrations of purified His6-GlnR in the binding buffer. Reaction mixtures were incubated for 30 min at 25°C, analyzed by electrophoresis using native 5% polyacrylamide gel run at 4°C with 0.5×TBE as running buffer, and electrophoretically transferred to a positively charged nylon membrane (GE healthcare, UK). Labelled DNAs were detected by chemiluminescence according to the manufacturer’s instructions, and recorded on X-ray film. The primers used here are listed in S6 Table.

### Chromatin immunoprecipitation-quantitative PCR (ChIP-qPCR)

Chromatin immunoprecipitation-quantitative PCR (ChIP-qPCR) was performed as described by [46]. 100 ml of culture of WT or Δ*glnR* grown in nitrogen-limited or -excess media at 30°C were harvested and immersed in cross-linked buffer (0.4 M sucrose, 1 mM EDTA, 10 mM Tris-Cl, pH 8.0) with 1% formaldehyde and 1% PMSF for 20 min at 28°C. Cross-linking was stopped by addition of glycine (final concentration 125 mM) and incubation for another 5 min. After cross-linking, cells were sonicated to break chromosomal DNA into 200–500 bp fragments. Supernatant containing 2 mg total protein was diluted in 1 ml lysis buffer. 5 μl GlnR polyclonal antibody was added into precleared supernatant and incubated overnight at 4°C. Purified immunoprecipitated DNA was resuspended in 120 μl double-distilled water. 2 μl DNA was used for qPCR, using the primer pairs listed in S6 Table. Relative levels of GlnR-coprecipitated DNAs were determined by comparison with negative controls.

### Surface plasmon resonance (SPR) detection

SPR experiments [47] were carried out using Biacore 3000 SPR sensor (Biacore AB, Uppsala, Sweden). All assays were carried out at 25°C. HBS buffer supplied with 3 mM MgCl_2_ (10 mM HEPES pH7.4, 300 mM NaCl, 3 mM MgCl_2_-6H_2_O, 0.2 mM EDTA, and 0.005% Tween-20) was used as the running buffer.

Protein-DNA interaction assays were performed with Sensor Chip SA. First, a biotinylated single-stranded DNA capture linker (biotin-GCAGGAGGACGTAGGGTAGG) was irreversibly bound to the chip. DNA oligomer used for SPR assays (S7 Table) were designed and synthesized based on *nif* promoter region harboring GlnR-binding sites and containing a single-stranded overhang complementary to the linker. Then a partially double-stranded DNA oligomer that contained the GlnR-binding site Ⅰ or site Ⅱ in the double-stranded region with a single-stranded overhang complementary to the capture linker was fixed onto the chip, reaching a signal of 250 RU. Control DNA was fixed onto flow cell 1 (FC1), and DNA containing GlnR binding sites were fixed onto flow cell 2 and 3 (FC2, FC3). GlnR with or without 25 nM GS protein was injected at a flow rate of 30 ul/min.

Protein-Protein interaction assays were performed with Sensor Chip CM5. GlnR was immobilized via amine groups onto all four flow cells, receiving a signal of 1000 RU. Purified GS with or without 1 mM glutamine were injected separately at a flow rate of 30 μl/min.

### Size-exclusion chromatography

Purified His_6_-GlnR from *E*. *coli* was used for size-exclusion chromatography. Analytical size-exclusion chromatography was carried out on an Akta purifier system equipped with a Superdex 200 column 10/300 (geometric column volume of 24 mL GE Healthcare). The running buffer contains 50 mM Tris-HCl (pH 7.4) and 300 mM NaCl. His_6_-tagged GlnR was diluted on running buffer to reach a concentration of 2 mg/ml. 1 mL filtered and centrifuged sample was injected at a flow rate of 0.3 ml/min. Purified His_6_-GlnR from *E*. *coli* was used for size-exclusion chromatography. Analytical size-exclusion chromatography was carried out on an Akta purifier system equipped with a Superdex 200 column 10/300 (geometric column volume of 24 mL GE Healthcare). The running buffer contains 50 mM Tris-HCl (pH 7.4) and 300 mM NaCl. His_6_-GlnR was diluted on running buffer to reach a concentration of 2 mg/ml. 1 mL filtered and centrifuged sample was injected at a flow rate of 0.3 ml/min. The apparent molecular weights of proteins were estimated after calibration of the column with standard proteins: thyroglobulin (670 kDa), globulin (158 kDa), ovalbumin (44 kDa), myoglobin (17 kDa), vitamin B12 (1.35 kDa) (Bio-Rad gel filtration standard).

### Acetylene reduction assays of nitrogenase activity

Acetylene reduction assays were performed as described previously to measure nitrogenase activity [12]. *P*. *polymyxa* WLY78 and its mutant strains were grown in 5 ml of LD media (supplemented with antibiotics) in 50 ml flasks shaken at 250 rpm for 16 h at 30°C. The cultures were collected by centrifugation, washed three times with sterilized water and then resuspended in nitrogen-deficient medium containing 2 mM glutamate containing 2 mM glutamate plus 0–100 mM NH_4_Cl as nitrogen source under anaerobic condition to a final OD600 of 0.2–0.4. Here, nitrogen-deficient medium containing 2 mM glutamate as nitrogen source and nitrogen-excess medium containing 2 mM glutamate and 100 mM NH4Cl as nitrogen source are generally used. Then, 1 ml of the culture was transferred to a 25-ml test tube and the test tube was sealed with robber stopper. The headspace in the tube was then evacuated and replaced with argon gas. After incubating the cultures for 6–8 h at 30°C with shaking at 250 rpm, C_2_H_2_ (10% of the headspace volume) was injected into the test tubes. After incubating the cultures for a further 3 h, 100 ml of culture was withdrawn through the rubber stopper with a gas tight syringe and manually injected into a HP6890 gas chromatograph to quantify ethylene (C_2_H_4_) production. The nitrogenae activity was expressed in nmol C_2_H_4_/mg protein/hr. All treatments were in three replicates and all the experiments were repeated three or more times.

### β-galactosidase assays

β-galactosidase activity was assayed according to the method described by [48]. Each experiment was performed in quintuplicate.

$$1\;unit\;of\;\beta -galactosidase=\frac{1000\times (OD_{420}-1.7OD_{550})}{Time(\min)\times vol(ml)\times \mathrm{Pr}.concentration\;(mg/ml)}$$ (1)

## Supporting information

S1 Fig General scheme of construction of deletion mutants. Suicide vector pRN5101 is used. Two DNA fragments upstream and downstream the target gene were PCR amplified from the genome DNA of *P*. *polymyxa* WLY78 and then were assembled to vector pRN5101 digested with appropriate restriction enzymes. Then the assembled product was transformed to *P*. *polymyxa* WLY78 and the double-crossover transformants were selected. Mutants Δ*glnR*, Δ*glnA*, Δ*glnA1*, Δ*glnRA* and MPnif97 (deletion of GlnR-binding site Ⅰ) were constructed in this way.
(PDF)

S2 Fig Comparison of growth of the wild-type (WT) and Δ*glnR* strains. Growth of the wild-type (WT) and Δ*glnR* strains in minimal medium supplemented with 30 mM glutamine (Gln) or glutamate (Glu) or NH_4_Cl as the sole nitrogen source.
(TIF)

S3 Fig Alignment of *P*. *polymyxa* GlnR with GlnR and TnrA of *B*. *subtilis* 168. The residues conserved among three proteins are indicated by white letters on a black background. The residues conserved between two proteins are indicated by black letter on a light grey background. A graphical representation of secondary structural elements is shown below the aligned sequences, where α-helices and β-strands are depicted as cylinders and arrows respectively. These secondary structural predictions except the Helix in the C-terminal domains were performed by using the PSIPRED server as described by McCuffin et al., 1990. The Helix in the C-terminal domains of the three proteins is predicted based on the analysis of the protein sequences described by Wray and Fisher, 2008.
(TIF)

S4 Fig Size-exclusion chromatography of *P*. *polymyxa* WLY78 His6-GlnR. **A.** The elution profile of P. polymyxa WLY78 His6-GlnR. Red line for markers, black line for His6-GlnR, signalling with arrows the two maxima. **B.** SDS-PAGE analysis of His6-GlnR. Lane 1: molecular weight markers (masses on the side, in kDa); Lanes 2 and 3, samples from the first and second maxima, repectively; Lane 4: Sample before application to the size-exclusion column.
(TIF)

S5 Fig Comparison of the inverted repeat sequences of GlnR/TnrA-binding sites. This is a graphic representation of the consensus sequences of GlnR/TnrA-binding sites of *Bacillales*, *B*. *subtilis* and *P*. *polymyxa* WLY78.
(TIF)

S6 Fig Comparison of GlnR-binding sites in the *nif* promoter region of *P*. *polymyxa* WLY78 and *P*. *riograndensis*. (TIF)

S1 Table Bacterial strains and plasmids used in this study. (DOCX)

S2 Table Primers used for construction of *gln* mutants, and their complementation and overexpression strains. (DOCX)

S3 Table Three 313 bp synthesized DNA fragments containing mutated GlnR-binding site (s). (DOCX)

S4 Table Primers used for mutation of GlnR-binding site in the *nif* promoter region of *P*. *polymyxa* WLY78. (DOCX)

S5 Table Primers used for overexpression of proteins in *E*. *coli* and P*nif*-*lacZ* fusion. (DOCX)

S6 Table Primers used for qRT-PCR, ChIP-qPCR and EMSA. (DOCX)

S7 Table Sequences of oligonucleotides used for SPR spectroscopy. (DOCX)

S1 Dataset (XLSX)
