# Supplementary figures and images for: Positive and negative regulation of transferred nif genes mediated by indigenous GlnR in Gram-positive Paenibacillus polymyxa
Source: PLoS Genet. 2018 Sep 28;14(9):e1007629. doi: 10.1371/journal.pgen.1007629 (PMC6191146; doi:10.1371/journal.pgen.1007629)

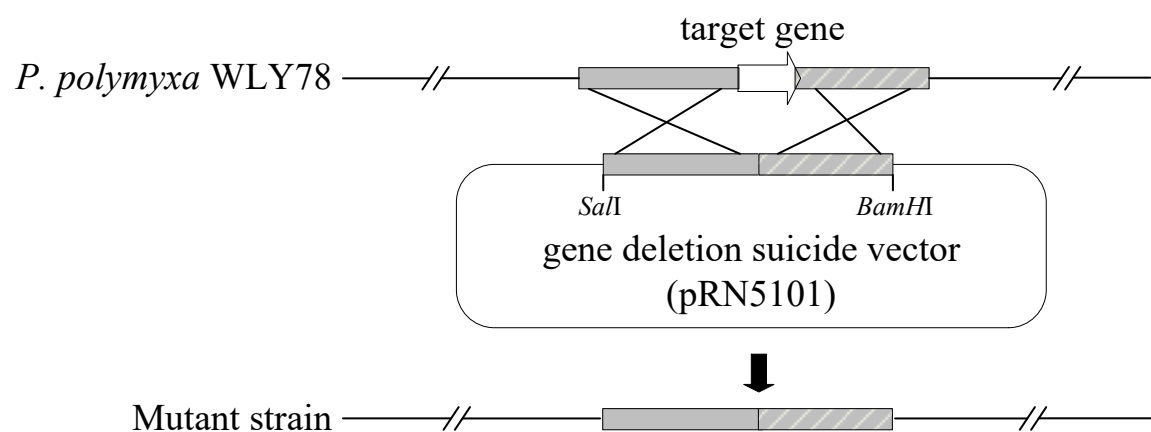

Supplement: S1 Fig — Suicide vector pRN5101 is used. Two DNA fragments upstream and downstream the target gene were PCR amplified from the genome DNA of P. polymyxa WLY78 and then were assembled to vector pRN5101 digested with appropriate restriction enzymes. Then the assembled product was transformed to P. polymyxa WLY78 and the double-crossover transformants were selected. Mutants ΔglnR, ΔglnA, ΔglnA1, ΔglnRA and MPnif97 (deletion of GlnR-binding site Ⅰ) were constructed in this way. (PDF) [file pgen.1007629.s001.pdf]

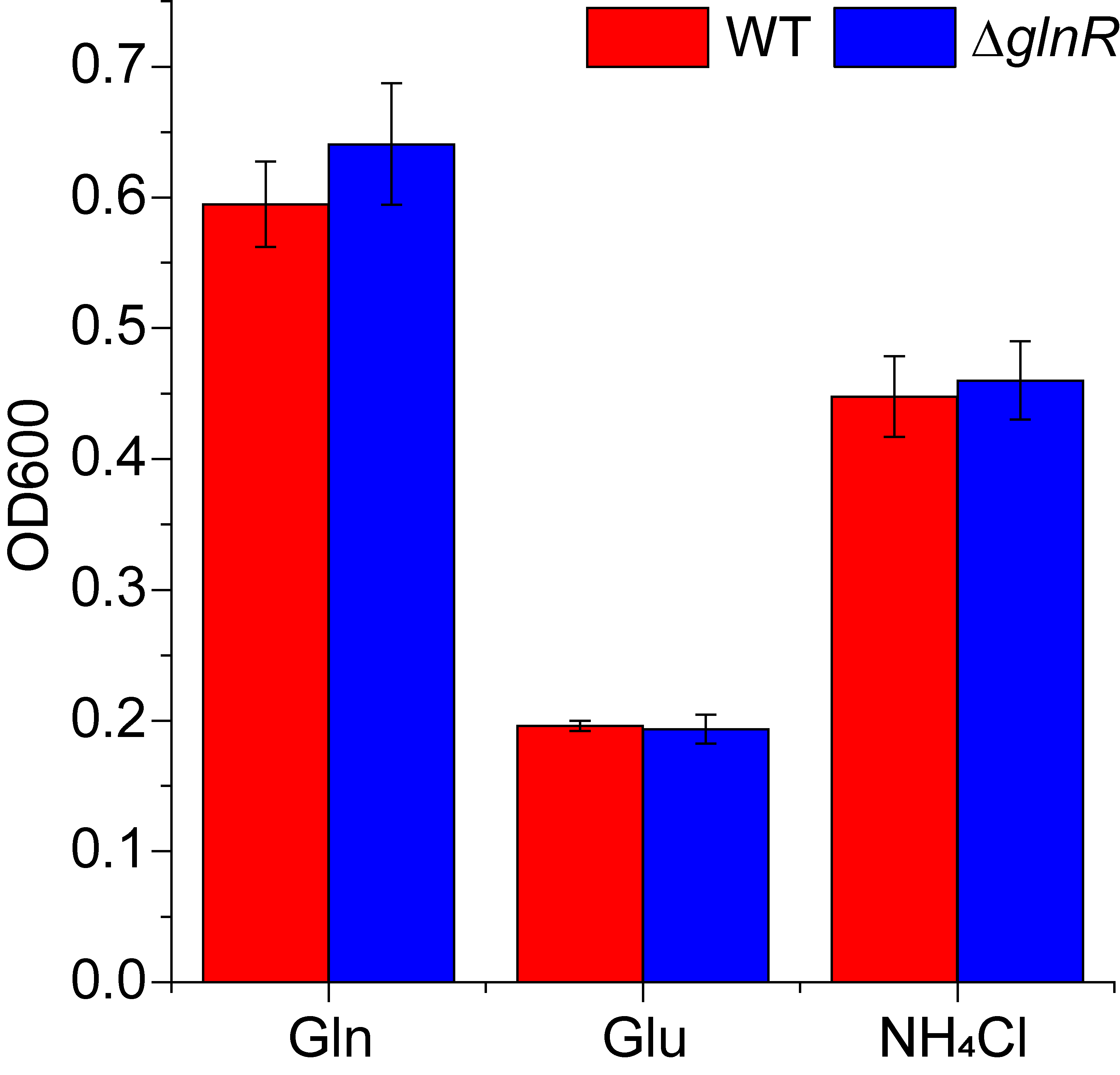

Supplement: S2 Fig — Growth of the wild-type (WT) and ΔglnR strains in minimal medium supplemented with 30 mM glutamine (Gln) or glutamate (Glu) or NH4Cl as the sole nitrogen source. (TIF) [file pgen.1007629.s002.tif]

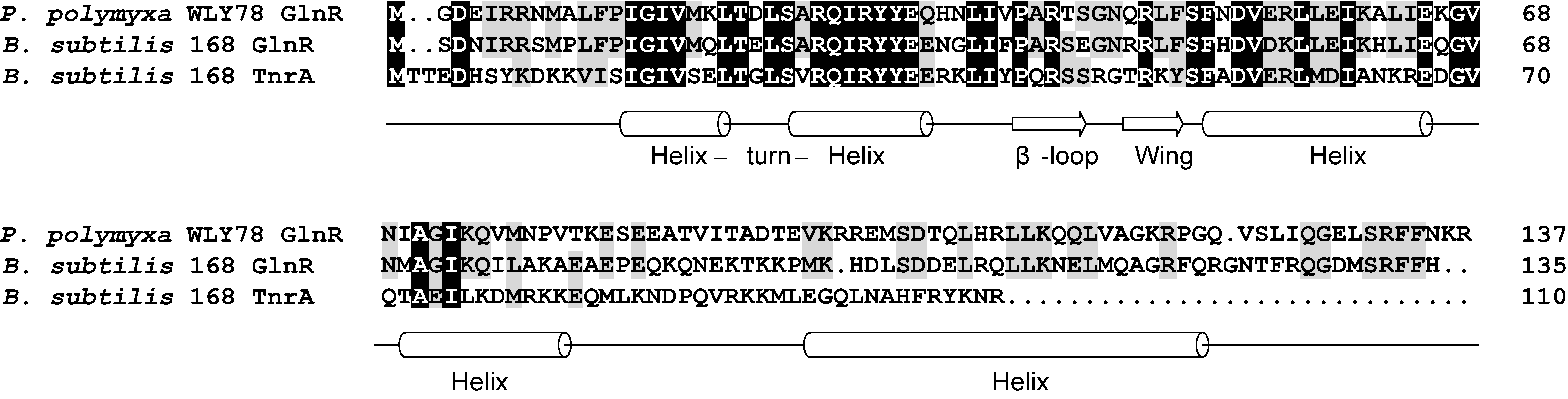

Supplement: S3 Fig — The residues conserved among three proteins are indicated by white letters on a black background. The residues conserved between two proteins are indicated by black letter on a light grey background. A graphical representation of secondary structural elements is shown below the aligned sequences, where α-helices and β-strands are depicted as cylinders and arrows respectively. These secondary structural predictions except the Helix in the C-terminal domains were performed by using the PSIPRED server as described by McCuffin et al., 1990. The Helix in the C-terminal domains of the three proteins is predicted based on the analysis of the protein sequences described by Wray and Fisher, 2008. (TIF) [file pgen.1007629.s003.tif]

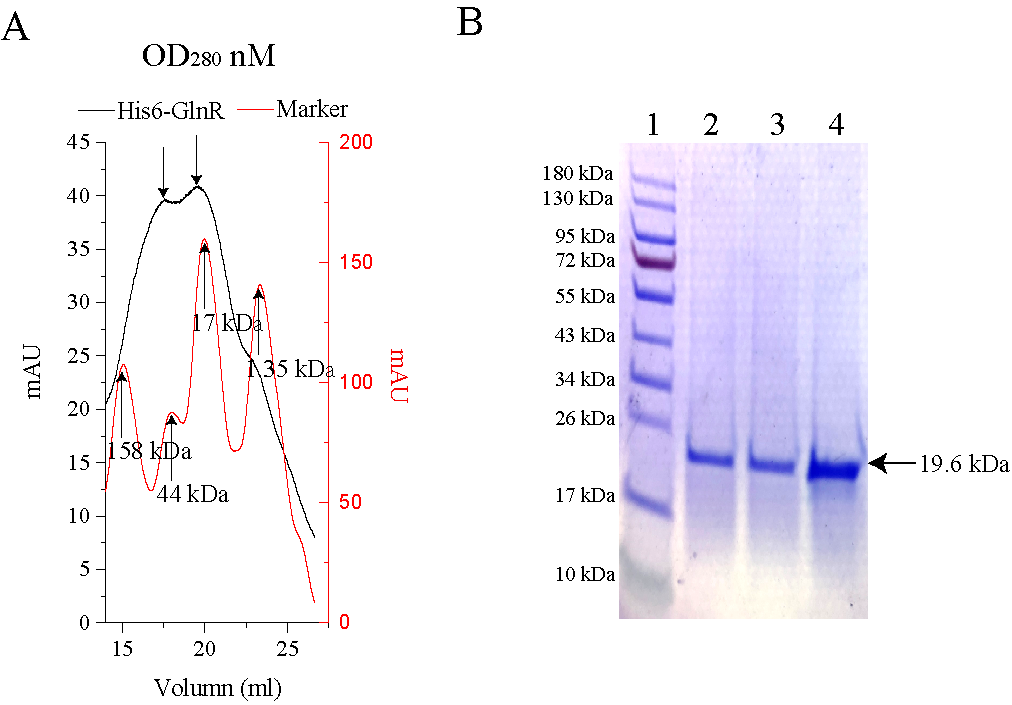

Supplement: S4 Fig — A. The elution profile of P. polymyxa WLY78 His6-GlnR. Red line for markers, black line for His6-GlnR, signalling with arrows the two maxima. B. SDS-PAGE analysis of His6-GlnR. Lane 1: molecular weight markers (masses on the side, in kDa); Lanes 2 and 3, samples from the first and second maxima, repectively; Lane 4: Sample before application to the size-exclusion column. (TIF) [file pgen.1007629.s004.tif]

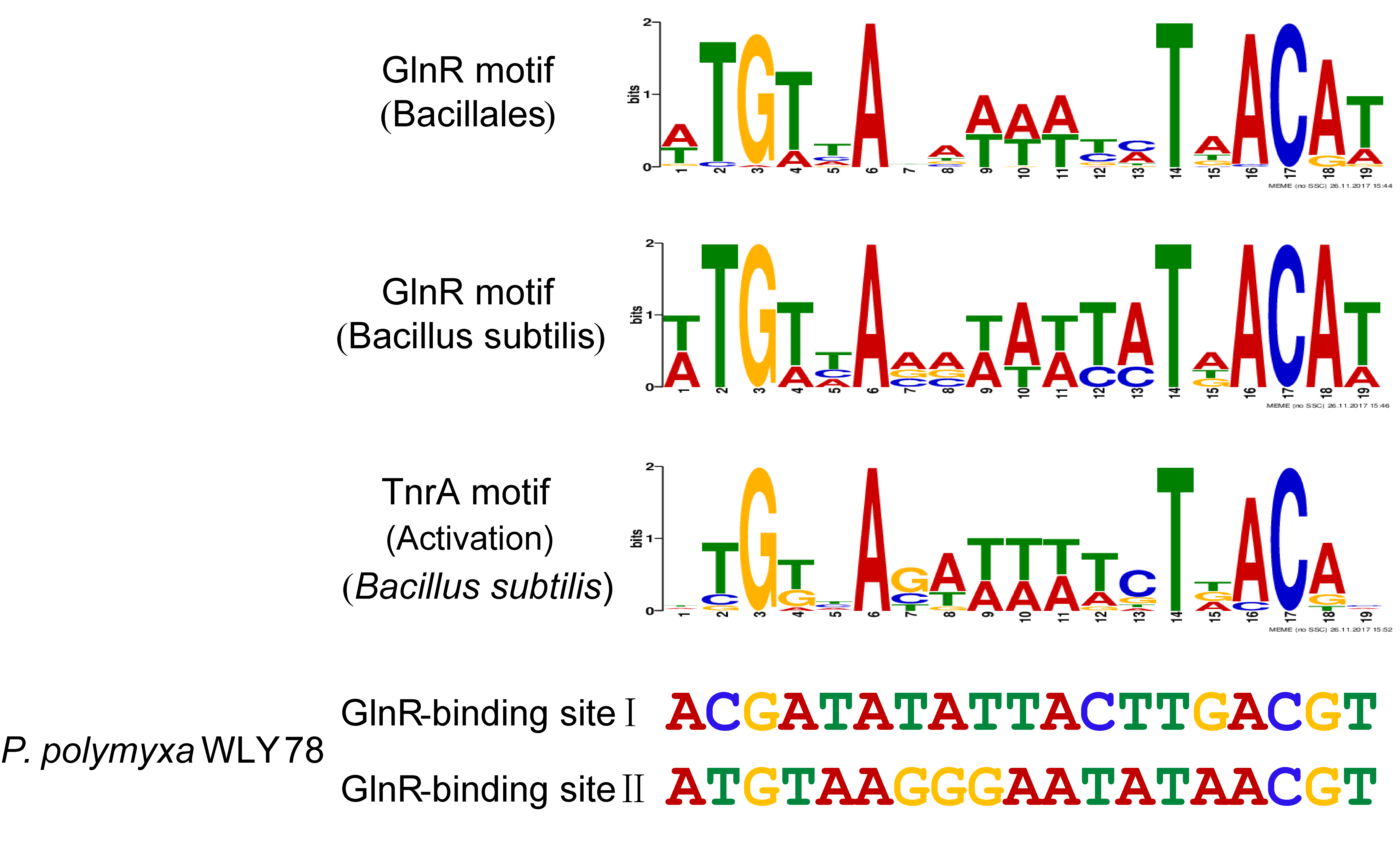

Supplement: S5 Fig — This is a graphic representation of the consensus sequences of GlnR/TnrA-binding sites of Bacillales, B. subtilis and P. polymyxa WLY78. (TIF) [file pgen.1007629.s005.tif]

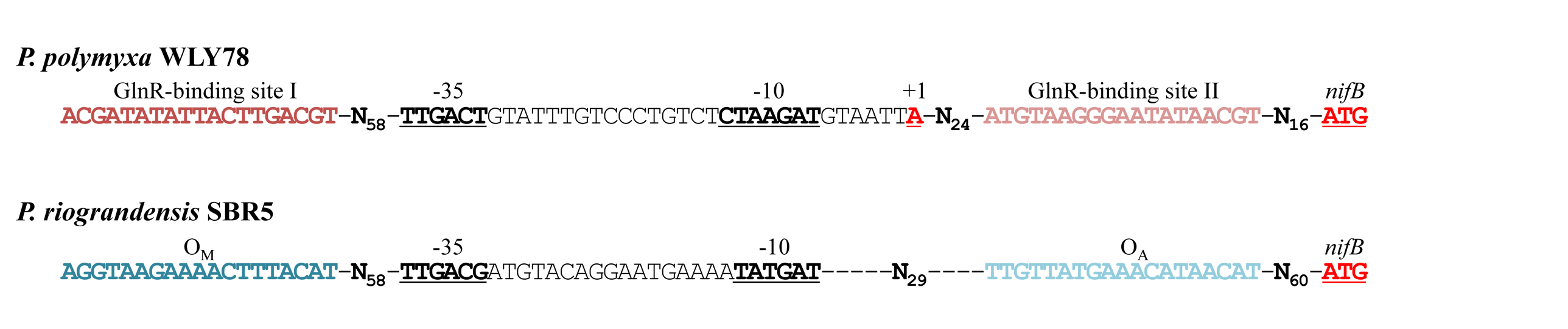

Supplement: S6 Fig — (TIF) [file pgen.1007629.s006.tif]
