# Supplementary material for: Positive and negative regulation of transferred nif genes mediated by indigenous GlnR in Gram-positive Paenibacillus polymyxa
Source: PLoS Genet. 2018 Sep 28;14(9):e1007629. doi: 10.1371/journal.pgen.1007629 (PMC6191146; doi:10.1371/journal.pgen.1007629)
Supplement: S1 Table — (DOCX) [file pgen.1007629.s007.docx]

| Strain or plasmid | Description | Source or reference |
| --- | --- | --- |
| *Paenibacillus polymyxa* | | |
| WLY78 | Wild-type strain | Laboratory stock |
| Δ*glnR* | *glnR* in-frame deletion mutant | This study |
| Δ*glnA1* | *glnA1* in-frame deletion mutant | This study |
| Δ*glnA* | *glnA* in-frame deletion mutant | This study |
| Δ*glnRA* | *glnRA* in-frame deletion mutant | This study |
| Δ*glnR/glnR* | Complementation strain of Δ*glnR* with *glnR* integrated on *amyE* locus as a single copy | This study |
| WT/*glnR* | *glnR* overexpression strain which is a derivative of *P. polymyxa* WLY78 carrying a *glnR* gene in plasmid pHYglnR | This study |
| Δ*glnA/glnA* | Complementation strain of Δ*glnA* with *glnA* carried in plasmid pHYglnA | This study |
| Δ*glnRA/glnRA* | Complementation strain of Δ*glnRA* with *glnRA* carried in plasmid pHYglnRA | This study |
| MPnif1 | A derivative of *P. polymyxa* WLY78 with site-specific mutation at GlnR-binding siteⅠ | This study |
| MPnif2 | A derivative of *P. polymyxa* WLY78 with site-specific mutation at GlnR-binding siteⅡ | This study |
| MPnif3 | A derivative of *P. polymyxa* WLY78 with site-specific mutations at both GlnR-binding sites | This study |
| MPnif97 | A derivative of *P. polymyxa* WLY78 with deletion of GlnR-binding siteⅠ | This study |
| *E. coli* | | |
| JM109 | General cloning host*; recA1, endA1, gyrA96, thi-1, hsdR17, supE44, relA1, Δ(lac-proAB*)/F’[*traD36, proAB+, lacIq, lacZ*ΔM15] | Sangon Biotech Co. |
| BL21 (DE3) | Host for protein overexpression; F^–^, *ompT, gal, dcm, lon, hsdSB*( *r_B_^-^m_B_*^-^-), λ (DE3 [*lacI, lacUV5-T7 gene 1, ind1, sam7, nin5*]) | Sangon Biotech Co. |
| Plasmids | | |
| pHY300PLK | Multiple-copy *E. coli-Bacillus* shuttle vector, *Tet*^R^ | TaKaRa |
| pRN5101 | Temperature-sensitive *E. coli-Bacillus* shuttle vector, *Ery*^R^ | (2) |
| pET-28b | Vector for His_6_-tagged protein overexpression in *E. coli*, T7 promoter and T7 terminator, pBR322 origin, *Kan*^R^ | Novagen |
| pRDglnR | *glnR* deletion vector based on pRN5101 | This study |
| pRDglnA1 | *glnA1* deletion vector based on pRN5101 | This study |
| pRDglnA | *glnA* deletion vector based on pRN5101 | This study |
| pRDglnRA | *glnRA* deletion vector based on pRN5101 | This study |
| pRCglnR | *glnR* complemented vector with *glnR* in pRN5101 | This study |
| pHYglnR | *glnR* overexpression vector with *glnR* in pHY300PLK | This study |
| pHYglnA | *glnA* complemented vector with *glnA* in pHY300PLK | This study |
| pHYglnRA | *glnRA* complemented vector with *glnRA* in pHY300PLK | This study |
| pRMP1 | A derivative of vector pRN5101 for mutation of GlnR-binding siteⅠ | This study |
| pRMP2 | A derivative of vector pRN5101 for mutation of GlnR-binding siteⅡ | This study |
| pRMP3 | A derivative of vector pRN5101 for mutation of both GlnR-binding sites | This study |
| pRMP97 | A derivative of vector pRN5101 for deletion of GlnR-binding siteⅠ | This study |
| pET*glnR* | *glnR* overexpression vector based on pET-28b | This study |
| pET*glnR*-25 | Vector for over expression of GlnR with 25 amino acids deleted based on pET-28(b) | This study |
| pET*glnA1* | *glnA1* overexpression vector based on pET-28b | This study |
| pET*glnA* | *glnA* overexpression vector based on pET-28b | This study |
| pHLP*nif* | P*nif*-*lacZ* fusion vector based on pHY300 PLK | This study |
