# Supplementary material for: Positive and negative regulation of transferred nif genes mediated by indigenous GlnR in Gram-positive Paenibacillus polymyxa
Source: PLoS Genet. 2018 Sep 28;14(9):e1007629. doi: 10.1371/journal.pgen.1007629 (PMC6191146; doi:10.1371/journal.pgen.1007629)
Supplement: S2 Table — (DOCX) [file pgen.1007629.s008.docx]

| **Gene name/ Location** | **Forward primer (5' - 3')** | **Reverse primer (5' - 3')** | **Target** |
| --- | --- | --- | --- |
| Upstream of *glnR* | glnRUF: AAGGCTCTCAAGGGCATCGGTCG  ACGCTTTATTTGGTGTGCTG | glnRUR: TTCACCTTGGGCCCATTTGTCATC  AACC | In-frame deletion of *glnR* |
| Downstream of *glnR* | glnRDF: ACAAATGGGCCCAAGGTGAATTAT  CACG | glnRDF: GCGACCACACCCGTCCTGTGGATC  CGCAGTGCATACCAGAACC |  |
|  |  |  |  |
| Upstream of *glnA* | up(*glnRA*)-F: CTCAAGGGCATCGGTCGACG  CGTCAGATATTGATCACG | up(*glnA*)-R: AGGAAAATGGGCTGGTATTCT  GAAACATG | In-frame deletion of *glnA* |
| Downstream of *glnA* | down(*glnA*)-F: TACCAGCCCATTTTCCTCTTT  CGCAATC | down(*glnA*)-R: CACACCCGTCCTGTGGATCC  TTGAGACCAAACCTGCCTG |  |
|  |  |  |  |
| Upstream of *glnR* | up(*glnRA*)-F: CTCAAGGGCATCGGTCGACG  CGTCAGATATTGATCACG | up(*glnRA*)-R: GGCGACGAGGCTGGTATTCT  GAAACATG | In-frame deletion of *glnRA* gene |
| Downstream of *glnA* | down(*glnRA*)-F: TACCAGCCTCGTCGCCCAT  TTGTCAT | down(*glnRA*)-R: CACACCCGTCCTGTGGATC  CTGCTTTATTTGGTGTGCTGC |  |
|  |  |  |  |
| Upstream of *glnA1* | up(*glnA1*)-F: ACGATGCGTCCGGCGTAGAG  GATCCTGTGCCGTTGAGTCTGCT | up(*glnA1*)-R: CACGGATGTTTCCTGAATTGT  TTTCAATACG | In-frame deletion of *glnA1* gene, |
| Downstream of *glnA1* | down(*glnA1*)-F: TTCAGGAAACATCCGTGTT  CATCCACAC | down(*glnA1*)-R: CGCAAAAGACATAATCGA  TAAGCTTCCGCCTTCTTCCTGAATC |  |
|  |  |  |  |
| Upstream of *amyE* | CglnR-amyE1: CGGCCACGATGCGTCCGGC  GTAGAGGATCCGTTGTGGTAGGTGCATACG | CglnR-amyE2: ATGCTCTGGTCCAGTATTTAT  CCGCTTCCTGG | Complementation of Δ*glnR* with *glnR* gene integrated on the chromosomal *amyE* locus as a single copy |
| Downstream of *amyE* | CglnR-amyE5: GTATAAGGTTGCCGCAATGC  TCAAATCAACTC | CglnR-amyE6: GACTGCGCAAAAGACATAAT  CGATAAGCTTATTCATACAAGCCGCTCC |  |
| *glnR* gene and its promoter | CglnR-amyE3: AGCGGATAAATACTGGACCA  GAGCATCTAATTG | CglnR-amyE4: ATTTGAGCATTGCGGCAACC  TTATACCAAGAG |  |
|  |  |  |  |
| Promoter region of *glnRA* operon | SR(*Sal*I): TATGTCGACAGTTATCATGGCTGCA  GGTA | SRP(*kpn*I): TACGGTACCATTTCTGCGAATTT  CGTCG | Complementation of Δ*glnA* with *glnA* gene in vector pHY300PLK |
| *glnA* ORF | SA(*kpn*I): TACGGTACCTGCCTTACTTTAAAA  CCTGG | SA(*Bam*HI): CCGGGATCCTTAGTAAAGCGTC  AGATATT |  |
|  |  |  |  |
| *glnRA* promoter and *glnRA* gene | SR(*Sal*I): TATGTCGACAGTTATCATGGCTGCA  GGTA | SA(*Bam*HI): CCGGGATCCTTAGTAAAGCGTC  AGATATT | Complementation of Δ*glnRA* with *glnRA* gene |
| *glnR* | CglnR1: TATGAATTCTGGACCAGAGCATCTA  ATTG | CglnR2: TATAAGCTTGGCAACCTTATACCAA  GAGGT | Overexpression of *glnR* gene in *P. polymyxa* WLY78 |
