# Supplementary material for: Positive and negative regulation of transferred nif genes mediated by indigenous GlnR in Gram-positive Paenibacillus polymyxa
Source: PLoS Genet. 2018 Sep 28;14(9):e1007629. doi: 10.1371/journal.pgen.1007629 (PMC6191146; doi:10.1371/journal.pgen.1007629)
Supplement: S3 Table — (DOCX) [file pgen.1007629.s009.docx]

| **Name** | **Sequences** |
| --- | --- |
| PnifM1 | TAAGCGGAGACTATTTCCCAAAATATATAATAAAAAATTAAAGTTTCTTATCTCAAAAGGAGAGCCGTATTTACGGACTCTCT  Mutated GlnR-binding siteⅠ  TTTTTTACGTCTTGATGTTATTGAGAATATGAAATGTAACCGCGCACATGTAAAGTGTACGATATATTACTGGTACCAAAATT -35 -10  TGACACATATGTGAATTGAGGATAAATGTCAGGGATTTCATGGAGAAGTGAA**TTGACT**GTATTTGTCCCTGTCT**CTAAGAT**G  +1 GlnR-binding siteⅡ Translation start of *nifB*  TAATT**A**TATTCCAGACAAAAACAGAGATTTATGTAAGGGAATATAACGTAGAGAGGAGGGAATGA |
| PnifM2 | TAAGCGGAGACTATTTCCCAAAATATATAATAAAAAATTAAAGTTTCTTATCTCAAAAGGAGAGCCGTATTTACGGACTCTCTTTTTTTACGTCTTGATGTTATTGAGAATATGAAATGTAACCGCGCACATGTAAAGTGTACGATATATTACTTGACGTAAAATTTGACACATATGTGAATTGAGGATAAATGTCAGGGATTTCATGGAGAAGTGAA**TTGACT**GTATTTGTCCCTGTCT**CTAAGAT**GT  Mutated GlnR-binding siteⅡ  AATTATATTCCAGACAAAAACAGAGATTTATGTAAGGGAATAATCGATAGAGAGGAGGGAATGA |
| PnifM3 | TAAGCGGAGACTATTTCCCAAAATATATAATAAAAAATTAAAGTTTCTTATCTCAAAAGGAGAGCCGTATTTACGGACTCTCT  Mutated GlnR-binding site Ⅰ  TTTTTTACGTCTTGATGTTATTGAGAATATGAAATGTAACCGCGCACATGTAAAGTGTACGATATATTACT**GGTACC**AAAATTTGACACATATGTGAATTGAGGATAAATGTCAGGGATTTCATGGAGAAGTGAA**TTGACT**GTATTTGTCCCTGTCT**CTAAGAT**GT  Mutated GlnR-binding site Ⅱ  AATTATATTCCAGACAAAAACAGAGATTTATGTAAGGGAATAATCGATAGAGAGGAGGGAATGA |

Red color: site-mutagenesis of GlnR-binding site (s)
