# Supplementary material for: Positive and negative regulation of transferred nif genes mediated by indigenous GlnR in Gram-positive Paenibacillus polymyxa
Source: PLoS Genet. 2018 Sep 28;14(9):e1007629. doi: 10.1371/journal.pgen.1007629 (PMC6191146; doi:10.1371/journal.pgen.1007629)
Supplement: S4 Table — (DOCX) [file pgen.1007629.s010.docx]

| **Gene name/ Location** | | **Forward primer (5' - 3')** | | **Reverse primer (5' - 3')** | **Target** |
| --- | --- | --- | --- | --- | --- |
| Upstream of *nif* promoter | | MPnif1: GCACGATGCGTCCGGCGTA  GAGGATCCGCCAACCGTGTAGACCGC | | MPnif2: TAGTCTCCGCTTATCATTCCT  TCACATCTATTTTCGTC | Mutation of GlnR-binding site (s) in *nif* promoter |
| Downstream of *nif* promoter | | MPnif5: GAGGAGGGAATGAATGGAC  TCTTTAGCTGATCTCTC | | MPnif6: CTGCGCAAAAGACATAATCG  ATAAGCTTCCACCACGACTAGCCAC |  |
| *nif* promoter region containing the mutagenesis of GlnR-binding site (s) | | MPnif3: TGTGAAGGAATGATAAGCG  GAGACTATTTCCC | | MPnif4: CTAAAGAGTCCATTCATTCCC  TCCTCTCTA |  |
|  |  | |  | |  |
| Upstream of *nif* promoter | | MPnif1: GCACGATGCGTCCGGCGTA  GAGGATCCGCCAACCGTGTAGACCGC | | MP100UR: AATACAGTCAATTTCATTC  CTTCACATCTATTTTC | Deletion of GlnR-binding siteⅠ |
| Truncation of *nif* promoter and its downstream | | MP100DF: GAAGGAATGATAAAATTG  ACTGTATTTGTCCCTGTCTCTAAGATG | | MPnif6: CTGCGCAAAAGACATAATCG  ATAAGCTTCCACCACGACTAGCCAC |  |
