# Supplementary material for: Positive and negative regulation of transferred nif genes mediated by indigenous GlnR in Gram-positive Paenibacillus polymyxa
Source: PLoS Genet. 2018 Sep 28;14(9):e1007629. doi: 10.1371/journal.pgen.1007629 (PMC6191146; doi:10.1371/journal.pgen.1007629)
Supplement: S5 Table — (DOCX) [file pgen.1007629.s011.docx]

| **Gene name/ Location** | **Forward primer (5' - 3')** | **Reverse primer (5' - 3')** | **Target** |
| --- | --- | --- | --- |
| *glnR* gene with His-tag | GlnRF: CGCGGATCCAATGGGCGACGAA | GlnRR: CCGCTCGAGTTATCTCTTATTGAA  GAACCG | Overexpression of His6-GlnR in *E. coli* |
| *glnR*^Δ25^, a deletion of 25 C-terminal codons | GlnRF: CGCGGATCCAATGGGCGACGAA | GlnR-25R: CCGCTCGAGTTGTTTCAGCAAGCG | Deletion of the C-terminal domain of GlnR |
| *glnA* gene with His-tag | GlnAF: TATGGATCCAAAGGGAGAGGTTAG  AGTGAGT | GlnAR: TATCTCGAGCGCCAAGGGATTTCC  CAT | Overexpression of His6-GS in *E. coli* |
| *glnA1* gene with His-tag | GlnA1F: TATGGATCCTTGGGAGGAAGAAG  AC | GlnA1R: TATCTCGAGTCAAAGCAGCTATG  CTA | Overexpression of His6-GS in *E. coli* |
| The *nif* promoter region | LPnif1: ACAGGAATTCCCGGGGATCCTAAG  CGGAGACTATTTCCC | LPnif2: TGTAAAACGACCATTCATTCCCTC  CTCTCTA | Construction of P*nif-lacZ* fusion |
| The *lacZ* gene from plasmid pPR9TT | LPnif3: GAGGAGGGAATGAATGGTCGTTTT  ACAACGTCGTG | LPnif4: GGAAAAACGCTTTGCCCAAGCTT  ATTTTTGACACCAGACCAAC |  |
