# Supplementary material for: Positive and negative regulation of transferred nif genes mediated by indigenous GlnR in Gram-positive Paenibacillus polymyxa
Source: PLoS Genet. 2018 Sep 28;14(9):e1007629. doi: 10.1371/journal.pgen.1007629 (PMC6191146; doi:10.1371/journal.pgen.1007629)
Supplement: S6 Table — (DOCX) [file pgen.1007629.s012.docx]

| **Gene name/ Location** | **Forward primer (5' - 3')** | **Reverse primer (5' - 3')** | **Target** |
| --- | --- | --- | --- |
| 16S rRNA | 16SF: TTTGTCGTCAGCTCGTGTTCGTG | 16SR: ATCCCCACCTTCCTCCGGTTTG | qRT-PCR |
| *nifH* | nifHF: ACCTGCCAGCTCTTCATACTC | nifHR: AACAGCCGGAATACGGACC | qRT-PCR |
| *nifD* | nifDF: TCATTCCTGTACGCTGTGAGG | nifDR: CACCGCCGATATTGTAGTCTC | qRT-PCR |
| *nifK* | nifKF: GCGGAGATGATTGCGGTATG | nifKR: GGCGTCATAGCCTGTAATATGTG | qRT-PCR |
| *glnR* | glnRF: AGCGCGTCAGATTCGTTATT | glnRR: CCTTCTCGATCAACGCCTTA | qRT-PCR |
| *glnA* | glnAF: TTAAGGAAGCCGAAGAGCTG | glnAR: TCGGAGCCAGGTCAAAATAC | qRT-PCR |
| GlnR-binding site Ⅰ | Pnifbox1F: ATATGAAATGTAACCGCGCACA | Pnifbox1R: CACTTCTCCATGAAATCCCTG  ACA | ChIP-qPCR |
| GlnR-binding site Ⅱ | Pnifbox2F: TTGACTGTATTTGTCCCTGTCTC  T | Pnifbox2R: GCGCAAAATAGCGATGTGCC | ChIP-qPCR |
| The probe of GlnR-binding site Ⅰ | EPnif1F: ACCGCGCACATGTAAAGTGTACGA  TATATTACTTGACGTAAAATTTGACACATATGTGA | EPnif1R: TCACATATGTGTCAAATTTTACGT  CAAGTAATATATCGTACACTTTACATGTGCGCGGT | EMSA |
| The probe of GlnR-binding site Ⅱ | EPnif2F: AGACAAAAACAGAGATTTATGTAA  GGGAATATAACGTAGAGAGGAGGGAATGA | EPnif2R: TCATTCCCTCCTCTCTACGTTATA  TTCCCTTACATAAATCTCTGTTTTTGTCT | EMSA |
| The probe of mutated GlnR-binding site Ⅰ | EPnif1MF: ACCGCGCACATGTAAAGTGTAC  GATATATTACTGGTACCAAAATTTGACACATATGTGA | EPnif1MR: TCACATATGTGTCAAATTTTGG  TACCAGTAATATATCGTACACTTTACATGTGCGCGGT | EMSA |
| The probe of mutated GlnR-binding site Ⅱ | EPnif2MF: AGACAAAAACAGAGATTTATGT  AAGGGAATAATCGATAGAGAGGAGGGAATGA | EPnif2MR: TCATTCCCTCCTCTCTATCGAT  TATTCCCTTACATAAATCTCTGTTTTTGTCT | EMSA |
