# Supplementary material for: Positive and negative regulation of transferred nif genes mediated by indigenous GlnR in Gram-positive Paenibacillus polymyxa
Source: PLoS Genet. 2018 Sep 28;14(9):e1007629. doi: 10.1371/journal.pgen.1007629 (PMC6191146; doi:10.1371/journal.pgen.1007629)
Supplement: S7 Table — (DOCX) [file pgen.1007629.s013.docx]

| **Oligonucleotide designation** | **LenLength ((ba(base)** | **Sequence (5' - 3')** | **Target** |
| --- | --- | --- | --- |
| BS1F | 39 | GTAAAGTGTACGATATATTACTTGACGTAAAATTTGACA | GlnR-binding site Ⅰ |
| BS1R | 59 | TGTCAAATTTTACGTCAAGTAATATATCGTACACTTTACCCTACCCTACGTCCTCCTGC |  |
| BS2F | 39 | AACAGAGATTTATGTAAGGGAATATAACGTAGAGAGGAG | GlnR-binding site Ⅱ |
| BS2R | 59 | CTCCTCTCTACGTTATATTCCCTTACATAAATCTCTGTTCCTACCCTACGTCCTCCTGC |  |
| NSF | 39 | CAGTGAGGCACCTATCTCAGCGATCTGTCTCAGTGAGGC | Nonspecific DNA |
| NSR | 59 | CAGTGAGGCACCTATCTCAGCGATCTGTCTCAGTGAGGCCCTACCCTACGTCCTCCTGC |  |
